# Current knowledge for implementing safe and sustainable by design across the life cycle of nanosilver textiles

**DOI:** 10.1007/s43621-025-02092-x

**Published:** 2025-12-03

**Authors:** Héctor D. Romero-Cantú, Irini Furxhi, Carla Thais Pereira Coelho, Ilaria Zanoni, Andrea Brigliadori, Anna Costa

**Affiliations:** 1 Department of Chemistry “Giacomo Ciamician”, Alma Mater Studiorum - University of Bologna, Via Selmi 2, 40126 Bologna, Italy; 2 ALBA Synchrotron, Carrer de la Llum, 2, 26, Cerdanyola del Vallès, 08290 Barcelona, Spain; 3 CNR-ISSMC, National Research Council of Italy-Institute of Science, Technology and Sustainability for Ceramics, Faenza, Italy; 4 Facultat de Química, Universitat de Barcelona, Barcelona, Spain; 5 Chemistry School of Natural and Environmental Sciences, Newcastle University, England, UK

**Keywords:** Silver nanomaterials, Textile applications, Eco-design, Green chemistry, Life-cycle thinking

## Abstract

Silver nanomaterials (AgNMs) are increasingly integrated into textiles due to their unique functionalities, such as antimicrobial, odour-resistant, and self-cleaning properties. Their widespread use, though beneficial, raises concerns about environmental and human health impacts across their entire life cycle spanning synthesis, manufacturing, use, and end-of-life. This review evaluates the scientific literature available, selected through structured keyword-based searches across major academic databases, focusing on studies relevant to AgNMs in textile applications. The analysis is framed by the 12 Green Chemistry Principles and the Safe-and-Sustainable-by-Design (SSbD) framework, a European policy initiative that promotes the integration of safety, sustainability, and functionality from early design stages. This review identifies the promising results of plant-based and enzymatic methods as safer and more sustainable options due to their reduced usage of toxic reagents and use of renewable sources. The manufacturing stage remains complex due to diverse integration methods, occupational exposure concerns, and inconsistent application of sustainability metrics. The use phase underscores environmental implications primarily from AgNM release during laundering, while direct human health risks remain comparatively low. Finally, the end-of-life stage reveals lower-than-anticipated ecological risks, largely due to effective sequestration of AgNMs in wastewater treatment sludges, although these demands a transition towards circular recovery strategies. The review highlights critical knowledge gaps in long-term environmental behaviour, exposure modelling, and standardization of performance metrics. Recommendations include expanding quantitative data collection, improving transparency in product labelling, and integrating circular recovery strategies. Advancements in SSbD frameworks, quantitative sustainability indicators, and EU-led initiatives provide actionable pathways to enhance stakeholder confidence and promote sustainable textile innovation. This work provides a comprehensive and policy-relevant synthesis to guide future research and safer innovation in nanomaterial-enabled textiles.

**Graphical abstract:**

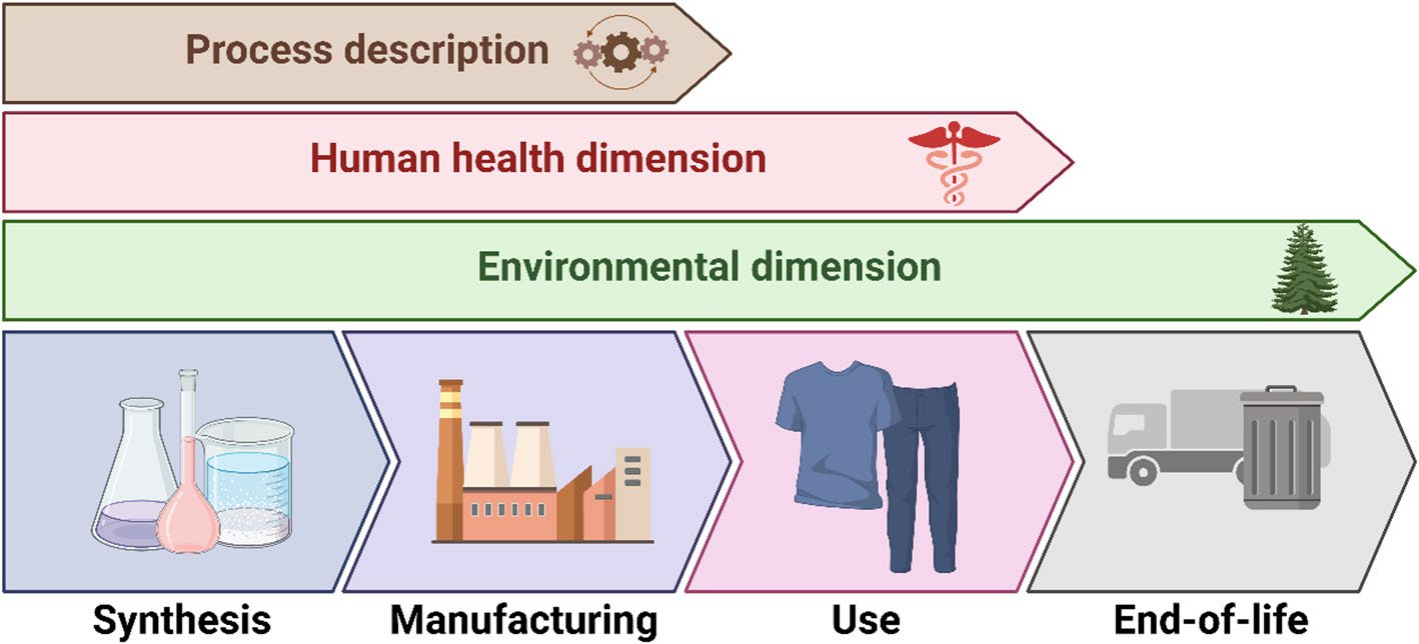

## Introduction

The rise of nanotechnology has led to the development of engineered nanomaterials (NMs) with enhanced chemical, physical, and mechanical properties compared to their bulk counterparts [1]. These materials now feature prominently across diverse applications—including textiles, where they offer improvements in durability, hygiene, and advanced functionalities like self-cleaning and antimicrobial performance [2–7]. Silver nanomaterials (AgNMs), in particular, have emerged as a preferred additive in textile applications due to their strong antimicrobial properties and versatility. Their integration can enhance dyeing, reduce odours, and support the development of smart textiles [8–11]. As of 2020, the global NMs market was valued at $10.34 billion, with AgNMs accounting for over 13% of the revenue [12, 13]. This market is projected to nearly quadruple by 2029, underscoring growing interest in these technologies.

Recently, AgNMs have been facing tightening of the European legislation which is limiting their employment as a biocide in different fields. Already in 2014, the Scientific Committee on Emerging and Newly Identified Health Risks (SCENIHR) of the European Commission (EC) expressed its opinion on safety, health and environmental effects and role in antimicrobial resistance of nano-silver with a particular focus on medical care and cosmetic products [14]. From the study, SCENIHR concluded that the widespread and increasing employment of AgNMs-containing products would lead to greater human exposure and environmental release of AgNMs and silver ions. At the time, the knowledge gap related to the risks associated with these phenomena required a more detailed investigation of the hazard/benefit profile of AgNMs as antimicrobial agents [15]. Over the years, this opinion has been updated several times such as in 2018 when the Scientific Committee on Consumer Safety (SCCS) of the EC expressed an updated opinion on colloidal silver (nano) limiting its concentrations in cosmetics including toothpaste and skin care products at 1% [16]. In 2021 the EC declared the non-approval of certain active substances in biocidal products. In particular, silver wasn’t approved as a disinfectant within product-type 2 “Disinfectants and algaecides not intended for direct application to humans or animals” and product-type 4 “Food and feed area” and as a preservative within product-type 9 “Fibre, leather, rubber and polymerised materials preservatives” [17]. Latest updates regarding the amendment of the cosmetic regulations in 2024. The SCCS deliberated that there is insufficient data to ensure nano-silver safety for cosmetic products representing a risk for consumer safety [18]. Because of AgNMs’ excellent antibacterial and antiviral activity, it’s still relevant to researchers, regulatory bodies and industry to study new solutions able to improve the hazard/benefit profile of AgNMs containing products along their full life cycle by: (i) developing new AgNMs synthesis, for instance using naturally-derived materials able to reduce Ag toxicity, (ii) identifying the best manufacturing approaches for the production of the nano-enabled product desired, such as a spray-coating process with an efficient suction system which minimizes wastes and workers exposure, (iii) enhancing the attachment of Ag to the product, in the case of textiles limiting user and environment exposure, and finally (iv) developing ad-hoc disposal procedures to avoid accidental release.

Decades of research on incorporating AgNMs into textiles have enabled the development of products with diverse characteristics, including variations in particle content, fabric type, and particle release. However, the industrial-scale production of these materials introduces new challenges and risks. Exposure to AgNMs can occur at any stage of the product life cycle, from synthesis and manufacturing to use and disposal [19]. Additionally, AgNMs release may impact environmental compartments, yet uncertainties remain regarding the environmental and human health effects of nano-enabled products (NEPs), particularly their (eco)toxicological mechanisms [20, 21]. In response to these concerns, a paradigm shift in material design has emerged. The EU Chemicals Strategy for Sustainability (CSS) introduced the SSbD concept, which integrates safety and sustainability considerations from the earliest stages of chemical, material, and product development [22]. Complementarily, the World Business Council on Sustainable Development (WBCSD) has developed a roadmap for the Chemical Industry Methodology for Portfolio Sustainability Assessments, aimed at guiding companies toward more sustainable product portfolios [23].

NanoReg2 project pioneered a step-by-step Safe-by-Design (SbD) implementation process [24, 25]. Subsequently, multiple Horizon Europe-funded initiatives have sought to define and validate SSbD implementations applicable across various sectors [26–30]. In 2022, the Joint Research Centre (JRC) published an SSbD framework for chemicals and materials, addressing four key dimensions—functionality, socio-economic sustainability, and environmental and health safety (EHS) considerations—across the product life cycle [31]. Ongoing case studies, including evaluations of plasticizers in food-contact materials, flame retardants in ICT products, and surfactants in textiles, continue to refine this framework [22, 31]. A primary challenge in the implementation of this framework is the paucity of data. In this context, the subsequent sections compile literature-based insights to capture the life cycle of Ag-coated textile production, encompassing the raw materials employed in AgNM synthesis, NEP manufacturing, product usage, and end-of-life considerations. This review examines SSbD interventions applicable at each stage of the textile life cycle, with a focus on reducing hazards, minimizing NMs release and exposure, and enhancing overall sustainability. We also take into account the 12 principles of green chemistry [32], which are regarded as a comprehensive summary of the achievements of green chemistry and a roadmap for advancements in the field, aligning chemistry with sustainability goals. Building on these frameworks, our structured synthesis of current knowledge and existing uncertainties across the textile life cycle offers a timely and constructive contribution, even where some aspects of the analysis remain qualitative in nature. This review uniquely integrates the principles of green chemistry with the regulatory-oriented SSbD framework across all life cycle stages of AgNM-enabled textiles, an approach that until now, has not been comprehensively addressed in previous literature.

## Methodology

The objective of the current review was to investigate the implementation of AgNMs across the textile life cycle through the lens of the SSbD framework. At each stage: (a) synthesis, (b) manufacturing, (c) use phase, and (d) end-of-life, we evaluated technological practices, human and environmental safety dimensions, and potential for alignment with the SSbD framework. Additionally, we assessed the relevance of each intervention in the context of the 12 Principles of Green Chemistry, aiming to identify practices that not only reduce intrinsic hazard and minimize release and exposure, but also enhance long-term sustainability.

To ensure a rigorous review process, a literature search was conducted using the databases Scopus, Web of Science, and ScienceDirect. The search was performed between December 2023 and February 2024, using the following Boolean combinations of keywords: “silver nanoparticles” OR “AgNMs” AND “textiles” AND “synthesis” OR “manufacturing” OR “use phase” OR “end-of-life” OR “life cycle” OR “SSbD” OR “green chemistry”. The time range was restricted to publications from January 2015 to December 2024, with preference given to studies published after 2020 to reflect recent advances. Inclusion criteria were: (i) peer-reviewed articles focusing on AgNM-enabled textiles; (ii) studies addressing at least one life cycle stage; (iii) literature providing experimental or review-based data on safety, environmental impact, or SSbD relevance. Exclusion criteria included non-English publications, conference abstracts without full papers and non-peer-reviewed commercial reports.

The selection of publications prioritized recent peer-reviewed studies, European regulatory reports, and technical guidelines addressing AgNMs in textiles. Articles were included based on their relevance to the life cycle stages, their integration of sustainability or safety principles, or their contribution to the advancement of SSbD concepts. Thematic emphasis was placed on processes that either mitigate or amplify risks at each life cycle stage, including emerging green synthesis approaches, occupational exposure scenarios, and leaching during product use.

Although no formal systematic protocol was applied, efforts were made to ensure comprehensive thematic coverage of both academic and regulatory perspectives. Rather than claiming an exhaustive coverage, this review aims to offer a structured synthesis of current knowledge, ordered within the scope of conceptual frameworks such as SSbD and the 12 Green Chemistry Principles. As such, the findings are intended to guide future research, regulatory actions, and industry practices toward safer and more sustainable innovation in nano-enabled textiles.

## Life cycle stages of AgNM-enabled textiles

In this review we have collected literature information and data regarding the implementation of AgNMs in textile industry, addressing each step of the life cycle of nano-silver coated textile: (a) synthesis stage, (b) manufacturing stage, (c) use phase, and (d) end of life. This analysis involves the processes employed and human safety and environmental dimensions and aims to identify the solutions that better aligns with SSbD framework evaluating pros and cons of different alternatives.

Since the same mechanisms proposed to justify the antimicrobial activity of AgNPs, such as membrane damage, ion release, and the production of reactive oxygen species, also pose a hazard to humans, it is essential to approach the development and large-scale production of nanoAg-enabled products according to the principles of SSbD [33]. In order to enhance the antibacterial and antiviral activity while minimizing risks to humans and the environment, it is necessary to study the entire life cycle of the material, implementing safe and sustainable solutions starting from the synthesis step, and evaluating the possible human exposure and environmental release pathways.

- Synthesis stage

Although some NMs occur naturally, AgNMs do not. The first life cycle step, i.e., the synthesis process takes place under controlled conditions to tailor their properties. This section outlines synthesis methods, their associated human and environmental risks, and alternative approaches aligned with the SSbD framework and/or the 12 principles of green chemistry.

i. Process

Multiple techniques are employed for AgNMs synthesis yielding distinct physicochemical characteristics. Among biological, physical, and chemical synthesis methods, the chemical methods are predominantly favoured due to their straightforward processing, scalability, and extensive documentation [34]. However, chemical processes must strive to align with green chemistry by preventing waste generation [GC Principle #1] and improving atom economy [GC Principle #2]. Physical methods, despite eliminating the use of chemical agents, are less frequently utilized because they often result in surface imperfections and poorly controlled particle morphologies [20].

Chemical reduction is the most widely applied chemical synthesis method in industrial settings due to its convenience, one-pot reaction design, and vast information in the literature [35, 36]. This process involves reducing Ag⁺ ions from a silver precursor in the presence of a reducing agent and a capping/stabilizing agent. Silver nitrate (AgNO_3_) is the preferred precursor due to its availability and solubility in polar solvents, though its potential environmental impacts necessitate evaluation of safer alternative chemicals [GC Principle #4]. The reducing agent facilitates the formation of silver atoms (Ag⁰), whereas the capping agent prevents excessive particle growth, ensuring nanoscale properties [37].

Commonly used reducing agents include *N*,* N*-dimethylformamide (DMF) [38], hydrazine [39], and ammonium formate [40]. However, these compounds pose health risks and are classified as potential carcinogens, raising occupational safety concerns [41, 42]. Such concerns underscore the importance of inherently safer chemistry for accident prevention [GC Principle #12]. Similarly, widely used stabilizing agents such as polyvinylpyrrolidone (PVP) [43], polyethylene glycol (PEG) [44], and ethylenediaminetetraacetic acid (EDTA) [45, 46] have raised concerns due to their potential bioaccumulation and persistence. Their degradation requires advanced treatment systems, complicating the transition toward sustainable alternatives and emphasizing the need for design-for-degradation strategies [GC Principle #10] [47, 48].

The increase in demand for green nanotechnology and chemistry has driven the adoption of green synthesis methods increasingly leverage renewable feedstocks [GC Principle #7], such as plant extracts and microorganisms, leading to reduced use of hazardous chemicals and waste generation [GC Principle #1 and #3]. Adopting green synthesis approaches not only improves public health and environmental quality but also offers socioeconomic benefits by reducing costs and creating employment opportunities, particularly beneficial in densely populated, resource-limited regions [49]. Furthermore, incorporating renewable biological resources enhances energy efficiency by enabling ambient synthesis conditions [GC Principle #6] and promoting catalytic bio-mediated processes instead of stoichiometric chemical reagents [GC Principle #9].

ii. Human safety dimension

While chemical reduction is a widely used method, many of the reagents involved present potential risks to human health emphasizing the need for inherently safer chemistry [GC Principle #12]. Early NM synthesis protocols rely on organic solvents and substances that either possess inherent toxicity or generate toxic by-products (e.g., DMF, hydrazine, PVP, PEG, EDTA). Roy et al. [50] demonstrated that these agents can alter the NM surface, influencing functionality and bioactivity. For example, AgNMs synthesized with toxic solvents such as DMF or PVP exhibit robust bactericidal properties but also substantial cytotoxicity, whereas those synthesized using phytochemicals maintain effective antimicrobial activity without the associated cytotoxic effects highlighting the importance of designing safer materials and processes [GC Principle #4, #3].

Driven by principles of green nanotechnology, green synthesis methods of AgNMs have been developed for the low-cost, non-toxic, and eco-friendly synthesis of highly biocompatible AgNMs, particularly for applications where toxicity is a critical concern [51, 52]. Utilizing renewable feedstocks, such as plant extracts, aligns well with green chemistry by avoiding hazardous substances [GC Principle #7], reducing waste generation [GC Principle #1], and lowering energy demand through ambient reaction conditions [GC Principle #6]. Among eco-friendly approaches, synthesis from plant extracts stands out as particularly suitable for both research and industrial applications [53]. Phytochemicals, acting simultaneously as reducing and stabilizing agents, have thus emerged as safer and more sustainable alternatives compared to conventional chemical reagents [35, 49, 54–56].

Studies have shown that AgNMs synthesized with tea extracts, polysaccharides, or epicatechin exhibit lower cytotoxicity compared to uncoated or conventionally synthesized particles, without compromising antimicrobial activity [49, 52, 54, 57, 58]. Biomolecule-based strategies involving glucose, cellulose, or starches further exemplify this approach [51, 54, 57, 59]. These compounds simultaneously act as reducing and stabilizing agents, minimizing auxiliary substances and unnecessary derivatizations [GC Principles #5 and #8], thus enhancing overall sustainability. While these alternatives may not always improve yield or reaction time, they offer a biocompatible solution while maintaining AgNMs’ desired functionality [49, 51, 52, 54, 57–59]. Table 1 provides a few examples of methodologies using chemicals with reported health concerns compared to green syntheses using naturally-derived compounds.

**Table 1:** Examples of AgNMs synthesis methods: comparison between more traditional approaches using concerning compounds vs. green synthesis using naturally-derived alternatives

Reducing agent	Capping/stabilising agent	Reaction time (h)	Additional assistance	Observations	References
*Traditional compounds*					
Hydrazine Hydrate (HH)	PVP	0.5	No	HH has been related to liver and kidney damage, additionally to its difficulty to handle. PVP is a biologically resistant polymer, thus potentially bioaccumulative.	[39]
DMF	3-(aminopropyl)tri methoxysilane	–	No	DMF is related to liver injuries, as well as increasing the risk of cancer.	[38]
NaBH_4_	EDTA	2	Heating (oil bath)	EDTA can be persistent and is related to eutrophication and potentially to the mineral absorption in animals and humans.	[45]
*Naturally-derived compounds*					
Plant extracts					
*L. acapulcensis* aqueous extract		0.25, 0.5, and 1	No	Same compound as reducing and capping agent, coming from a natural source. Not much filtration needed, no exact active ingredient mentioned.	[55]
*Citrus* L. aqueous extract		0.5	No	Same compound as reducing and capping agent. Natural source origin. No exact active ingredient mentioned.	[60]
Curcumin		–	No	Curcumin is approved as food additive. No relevant health risk associated to curcumin are reported. Several studies propose curcumin as natural remedy with presumed anti-inflammatory and antibacterial properties.	[61]
*Artemisa annua* L. extract		1	No	*Artemisia annua* L. has been identified as traditional herbal remedy against inflammatory disease, infections by bacteria and viruses.	[62]
*Sargassum muticum* macroalgae extract		0.5	Heating	*Sargassum muticum* is an aggressive and invasive brown macroalgae with antioxidant, antibacterial, anti-inflammatory and anti-obesity properties.	[63]
NaBH_4_	Ulvan polysaccharide powder (*Ulva armoricana* extract)	**–**	No	Natural source capping agent. Activity attributed to one specific compound (ulvan).	[64]
Purple cabbage anthocyanin extract (*Brassica oleracea var. capitata*)		0.25	No	Same compound used as reducing and capping agent. Activity attributed to one specific compounds (anthocyanins).	[65]
*Abundant biomolecules*					
Glucose		0.5	UV light (λ = 365 nm)	Glucose as both capping and reducing agent. Vast availability from a wide variety of sources	[66]
Pectin		1 and 2	Microwave	Pectin as both capping and reducing agent. Available in different natural sources, mainly fruit peels.	[67]
Hydroxyethylcellulose (HEC)		48	No	HEC as both capping and reducing agent. Product derived from cellulose, another abundant biomolecule in plants and some other species.	[51]
*Biogenic origin*					
*Pseudomonas aureginosa*		48	No	*Pseudomonas aeruginosa* is a bacterium which can cause infections in the blood, lungs, and skin.	[68]
*Setosphaeria rostrata* fungi		24	No	Endophytic fungi are source of secondary metabolites with antimicrobial, antioxidant, anti-cancerous, anti-diabetic and antiviral properties.	[69]

The synthesis process may pose human health risks due to potential exposure. AgNM have been linked to DNA damage, genotoxicity, and inflammatory responses in organs such as the liver and kidneys, along with functional impairments in the lungs, heart, intestines, and spleen. AgNMs can traverse biological barriers and enter systemic circulation [70, 71]. Furthermore, it has also been stated in several publications that AgNMs are biodistributed throughout the body, and that factors such as size, morphology, and capping agent might enhance their movement to different organs, despite being exposed through inhalation only [72]. Thus, although inhalation is particularly relevant in occupational settings, a portion of inhaled AgNMs is expected to be cleared via the gastrointestinal tract following mucociliary clearance.

The evaluation of AgNM toxicity requires comprehensive physicochemical characterization, as properties such as morphology, size, charge, coating, chemical composition, redox potential, dissolution, ion release, and aggregation substantially influence biological interactions [70]. Modifications to the NM surface, including coating and doping, can markedly alter both antimicrobial activity and toxicity. For instance, Motta et al. [33] demonstrated that surface doping using hydroxyethyl cellulose improved water dispersion—advantageous for manufacturing—but also enhanced particle–cell membrane interactions, increasing cytotoxicity against A549 cells. Further studies have pointed out that AgNMs with small diameters are distributed in organs such as brain, liver, kidney, and other organs, whereas particles with larger sizes were not that widespread [3] Similarly, research by Azizi-Lalabadi, et al. revealed that 4 nm AgNMs induced significant toxicity in U937 macrophage cells at 3.12 mg/mL, whereas 20 nm particles required 25 mg/mL to achieve comparable effects [73]. Toxicological assessments are indispensable for correlating the physicochemical characteristics of AgNMs with their biological interactions [72, 74]. Although considerable progress has been made in characterizing AgNMs, further research is necessary to understand their behaviour in complex matrices [75, 76].

Recent studies emphasize the importance of synthesizing AgNMs with controlled properties to ensure targeted functionalities and/or safety [77]. However, synthesis involves multiple parameters, including reaction duration, scale, and the choice of capping agents, all of which significantly affect properties. To address these challenges, Furxhi et al. [77] developed machine learning models using literature extracted data on synthesis conditions, physicochemical characteristics and antibacterial efficiency, and toxicological profiles. Regression algorithms enabled the prediction of NMs core size and antibacterial activity, with Shapley value analyses highlighting critical factors influencing synthesis outcomes. Complementarily, Furxhi et al. [78] developed quantitative intrinsic hazard criteria from FAIR (findable, accessible, interoperable, and reusable) data. Their study utilized Quantitative Structure-Activity Relationship (QSAR) models based on machine learning regression and classification to predict NM hazard classes. These models incorporated both system-dependent (e.g., hydrodynamic size, polydispersity index) and non-system-dependent (e.g., elemental composition, core size) properties, as well as biological and experimental conditions. Through expert-driven Bayesian network analyses, interpretable safety criteria were identified, achieving approximately 78% predictive accuracy. This provided guidance for synthesizing inherently safer NMs in a cost-effective in silico toxicological screening, for early-stage implementation of SSbD principles.

iii. Environmental dimension

A comprehensive evaluation of a material’s synthesis environmental impact must consider the full life cycle of its precursors and reagents aligning closely with green chemistry’s emphasis on pollution prevention [GC Principle #1]. For AgNM, the primary silver source is derived from ore, whose extraction incurs significant environmental repercussions. Quantified impacts such as greenhouse gas emissions, ionizing radiation, ozone formation and depletion, water depletion, particulate matter emissions, acidification, eutrophication, and resource depletion. Specifically, silver extraction contributes approximately 62.12 kg CO_2_-equivalent greenhouse gases, 0.18 kg PM_2.5_-equivalent particulate matter, and 36.697 kBq U-235-equivalent ionizing radiation, making it significantly more pollutant-intensive compared to metals such as zinc, copper, or lead [79]. Furthermore, most chemical synthesis routes require an Ag-containing compound (e.g., AgNO_3_, Ag_2_SO_4_, or AgClO_4_) [80]. In these processes, silver ores react with strong acids (HNO_3_, H_2_SO_4_, HClO_4_), generating hydrogen gas, a flammable by-product that, if not properly managed, poses explosion risks and compromises worker safety [GC Principle #12]. The emission of acidic gases, particularly HNO_3_ and H_2_SO_4_, also contributes to acid rain, which disrupts soil chemistry, causes mineral depletion, and adversely affects wildlife and human populations [81]. Given these impacts, the extraction of Ag and the subsequent transformation into precursor salts represent some of the most critical steps in the AgNM life cycle in terms of sustainability. Efforts to “green” AgNM synthesis aim to reduce both the environmental and safety impacts of traditional methods. Two main strategies have emerged:

*a. Improving synthesis efficiency through energy input*: In line with Jacobs et al. (2010)’s [82] principles for green nanotechnology, focusing on material and energy efficiency [GC Principles #2, #6], researchers have developed assisted synthesis methods that use additional energy sources (e.g., microwaves, ultrasounds, or electrical currents) [83–87] to enhance yields and shorten reaction times. For example, Su et al. (2019) [67] and Ashraf et al. (2020) [88] demonstrated that microwave or UV-assisted methods can reduce reduction times from 30 min to as little as 30 s. Similarly [89], reported a reduction in reaction time from 150 to 90 min. Despite improved efficiency, the additional energy input required could offset overall sustainability gains by increasing the carbon footprint, highlighting the need to carefully balance improved synthesis efficiency against energy consumption [GC Principle #6].

*b. Utilizing more potent reducing agents*: An alternative approach is to employ reducing agents with high radical scavenging capacity, such as anthocyanins, polyphenols, sugars, and polyols [90–93]. Often derived from renewable feedstocks like plant extracts, cellulose, or starches [GC Principle #7] [59], these compounds can serve dual roles as both reducing and stabilizing agents, thereby minimizing the number of reagents and auxiliary substances required [GC Principles #5 and #8]. The main advantage of this approach is that it offers a safer and more sustainable process while achieving yields comparable to those of conventional methods like those employing molecules such as DMF or NaBH_4_. For further illustration, Table 1 provides a comparative overview of greener AgNM synthesis methodologies in which hazardous chemical reagents are replaced with natural alternatives such as plant extracts, glucose, cellulose, and starches. Nonetheless, the extraction and purification of these bioactive compounds present their own challenges. However, extraction and purification of bioactive compounds currently rely on substantial quantities of organic solvents (e.g., acetone, methanol, ethanol, chloroform), which contradict the principle of using safer solvents and auxiliaries [GC Principle #5] [94, 95], as outlined by Anastas and Walker [32].

Plant-mediated green NM synthesis has monumental prospects for a sustainable future but necessitates continued research to determine its full potential. Part of these results are due to the high contents of bioactive chemicals and enzymes—like phenols, flavonoids and terpenoids. Despite advancements in the environmentally friendly production of AgNMs, several problems still exist, especially regarding the standardization of synthesis techniques, large-scale manufacturing, and thorough knowledge of the fundamental principles regulating NM formation [54]. For instance, Khane et al. [60] relied on UV-Vis peak intensities rather than quantitative yield percentages, while Antony et al. and Kumar et al. [96, 97] employed disparate metrics (e.g., inhibitory versus radical scavenging activity). Although each group succeeds in reporting a reaction yield, and thus claiming an effective synthesis, it remains uncertain whether calculating the yield through a different method would estimate the same. Therefore, the implementation of SSbD as a framework or reference, it would be important to define benchmark method for yield calculation, so that the overall impact of more sustainable and safer methods are defined with the least bias possible.

b. Manufacturing stage

Manufacturing refers to the process of incorporating materials into products or components using tools, human labour, chemical processes, and machinery. With the increasing industrial use of AgNMs, it is imperative to evaluate their environmental and human health impacts. To this end, a comprehensive understanding of the chemical and physical properties of the materials is essential [98].

i. Process

**Fig. 1:**
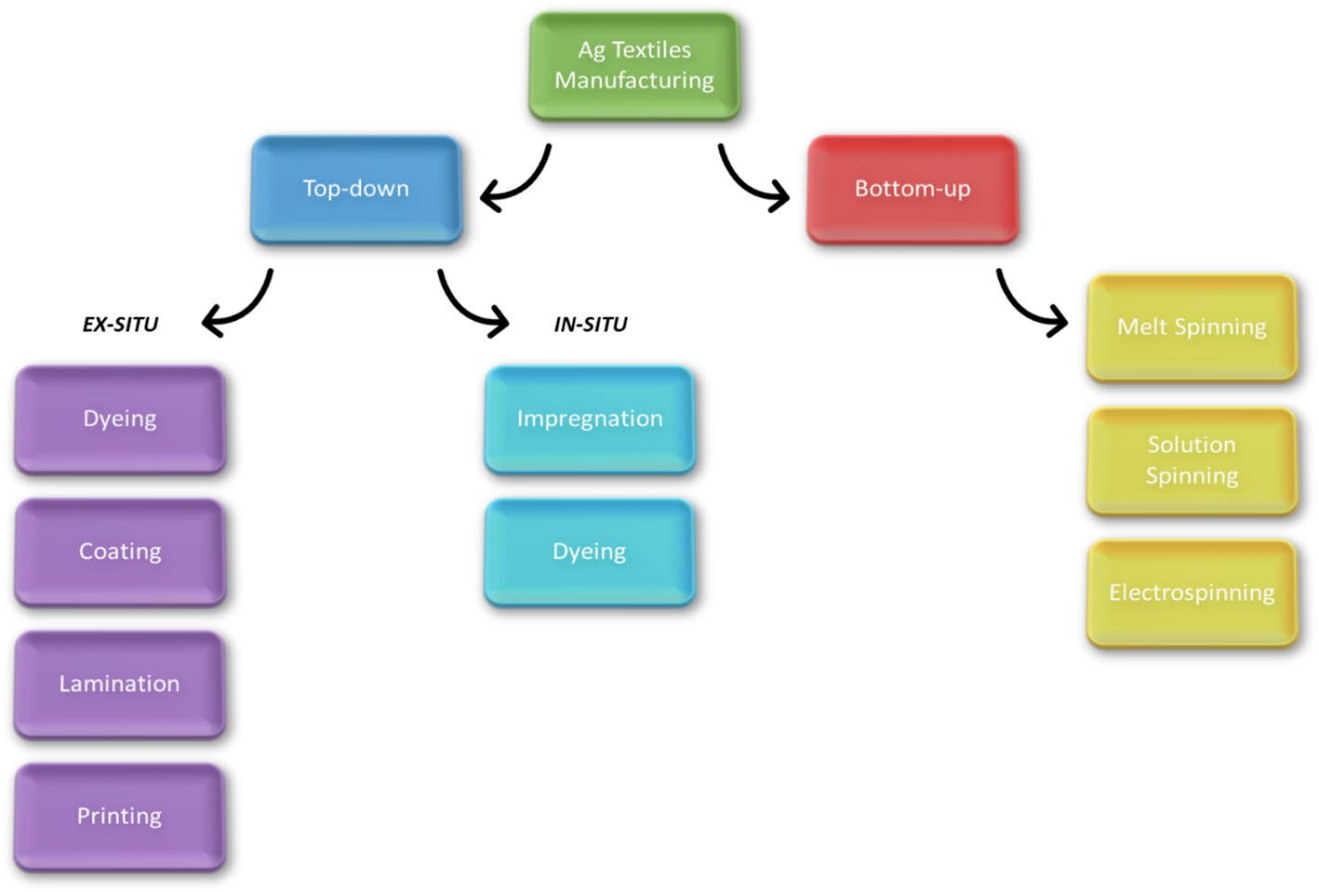
Bottom-up and top-down approaches to incorporate AgNMs into substrates

The conventional textile industry has achieved improvements in product performance—including enhanced mechanical strength, durability, fabric texture, and the ability to produce vibrant colours and intricate printing patterns [11]. Incorporating NMs into textiles results in wearable technology and smart textiles, which can react to external stimuli and offer additional functionality [99]. Various methods are employed to functionalize textiles with NMs; these methods either embed the particles within fibres or apply them as surface coatings. On an industrial scale, some of the methods used for the incorporation of NMs into textiles include particle impregnation, spray coating, multifunctional composite fibre drawing, and direct weaving [99]. (see Fig. 1)

Textile functionalization via fibres typically follows a “bottom-up” approach, while finishing steps adopt a “top-down” approach. Both approaches yield varying degrees of efficiency, NM leaching potential, and environmental impact, highlighting the importance of preventing pollution and minimizing hazardous by-products [GC Principle #1] (see Table 2).

**Bottom-up** approaches involve integrating AgNMs during fiber formation. This can be accomplished via processes such as melt spinning (where polymers are melted, extruded, and rapidly quenched), solution spinning (in which a polymer is dissolved, extruded, and the solvent is removed via evaporation, dry spinning, or wet spinning), or electrospinning. This latter employs a high-voltage electrostatic field to generate ultrafine fibers from a polymer solution that are deposited on a grounded collector [100]. Although electrospinning offers better control over fiber diameter, morphology, and functionality, its reliance on solvent recovery and the associated toxicity issues can compromise sustainability [101].

In contrast, top-down approaches apply NMs as a finishing step. These can be subdivided into: a. **In-situ methods**, where NMs are synthesized directly on fabric surfaces during treatment [102–104]; While offering scalability and mild processing conditions (temperatures below 100 °C), these methods can involve hazardous precursors and wastewater production, necessitating a safer chemical design [GC Principles #3 and #4]. To mitigate this, researchers explore substituting hazardous chemicals with renewable, plant-derived alternatives, aligning with green chemistry principles [105]. Jiang et al. [106] demonstrated a microwave-assisted synthesis using anisotropic Ag nanoplates, controlling shape and functionality, thus greening the dyeing process while incorporating antibacterial and gas-sensing properties [GC Principles #6 and #7].

b. **Ex-situ methods**, which involve the synthesis of NMs followed by their incorporation into the fabric via dyeing, coating, or printing [11, 103, 107]. Coating methods involve applying a NMs layer onto one or both sides of the fabric, followed by curing to promote adhesion [99]. Techniques include direct coating, spraying, dipping, foaming, transfer, calendering, and hot-melt extrusion. Lamination can also be used, depending on the resin’s rheological properties [107]. Within coating it is found the widely used pad-dry-cure method, which is simple and adaptable, though the drying stage could be energy- and time-intensive raising concerns over energy efficiency [21] [GC Principle #6]. Moreover, the exhaustion method requires precise control of variables such as temperature, pH, and NM concentration, making it less cost-effective than padding [107].

The two most popular methods for textile functionalisation are dip-coating and spray-coating. While dip-coating, consists of dipping the textile in the NM-containing solution, and then retiring it to allow it to dry [108], spray-coating makes use of atomizers to spray such solution onto the textile [109]. For both techniques, one of the most important aspects is coating thickness for it will confer the textile the sought properties. In order to achieve a known coating thickness, each technique considers different parameters. For instance, dip-coating considers parameters such as dipping time, surface morphology, textile substrate tension, temperature, withdrawal speed, compound concentration, coating bath composition, among others [108, 110]. Conversely, spray-coating considers some others, such as spray rate, atomization air pressure, inlet and exhaust air temperature, nozzle size, nozzle-to-bed distance and on-site control measures [109, 111]. Each of the techniques has its pros and cons. While dip-coating might be easier to adapt to existing technologies, the variance of smoothness/roughness of the layers, and their thickness might hinder their applications [108]. Spray-coating on the other hand, exhibits a controlled film thickness and roughness, minimal liquid waste, and the possibility of using a wide spectrum of different viscosity fluids [GC Principles #2, #5] [109, 112].

Nonetheless, NM-adhesion challenges persist, potentially increasing risks of environmental release [102, 107]. Stabilizers enhance NM fixation but selecting additives compatible with environmental sustainability remains critical [GC Principle #4] [113]. Various parameters and coating techniques can be adjusted to improve such factor. For instance, Igal et al. [114] demonstrated the advantages of dip-coating sol–gel technology for fabricating functionalized textiles incorporating C, Ag, and Zn to achieve antimicrobial activity. The method reported relies on hydrolysis, condensation, and polycondensation reactions to develop inorganic matrices from metal precursors such as silver, followed by their deposition to the textile material through dip-coating. This method represents a greener and more sustainable coating approach, as it operates at lower temperatures using eco-compatible synthesis protocols, and facilitates the formation of a selective oxidic network, improved durability, enhanced stability, and a cross-linked silica matrix on the textile surface, thus demonstrating inherently safer chemistry [GC Principles #4, #5, #6, and #12] [114].

Printing techniques including gravure, offset, flexography, pad, roll-to-roll, screen, inkjet, and slot-die printing offer additional routes for applying AgNMs to textiles. The development of high-performance inks is critical to ensure comfort, flexibility, washability, and NM stability [115]. Printing is particularly effective for mass-producing smart textiles, as it enables versatile patterning with minimal waste [GC Principle #1 and #2], facilitating mass production of smart textiles. Ensuring robust NM fixation remains challenging; however, post-treatment strategies (e.g., UV-curing) significantly enhance structural integrity and environmental stability, contributing to safer chemistry and reduced NM leaching [GC Principles #4, #12] [107, 115, 116].

**Table 2:** Comparison of AgNMs incorporation methodologies for nano-enabled products manufacturing, advantages and disadvantages

Method	Technique	Advantages	Disadvantages	References
*Bottom-up*				
Spinning techniques	Solution spinning	Permit the functionalization of matrices without stable melt phase and with different properties. ***Diverse functionalization***	The structure and properties depend on the polymer-solvent interactions, and the productivity is low, so the manufacturing needs more equipment, making the process more expensive. Organic solvents are needed in the process. ***Costly and less sustainable***	[107]
	Melt spinning	Relatively simpler and cheaper than solution spinning; due to high productivity, ease to spin multicomponent fibers with different cross-sectional shapes, and no addition of toxic solvents. ***Cheap and sustainable***	Nonuniformity of diameter, thermal decomposition during extrusion and use of thermoplastic polymers. ***Limited applications***	[107]
	Electrospinning	Simple and low-cost compared to melt and solution spinning. Also, it allows control of the fibre diameter, morphology, and functionality, and has a mass-production potential. Simple and low-cost method.	Regarding sustainability, the drawbacks are related to solvent recovery and toxicity. Sustainability concerns, focused on the solvents	[117]
*Top-down*				
Dyeing	Exhaustion	Many kinds of textiles can be functionalized by this process, such as fibres, yarns, knitted and woven fabrics, ***Diverse functionalization***	It requires precise control of the conditions, such as temperature, time, pH, NMs concentration, and addition of supplementary agents. ***Complex technique.*** ***Low sustainability.***	[118]
	Padding	Allows the functionalization of large fabric portions, which is simple, productive, low maintenance and cost-effective. Diverse functionalisation. ***High productivity.*** ***Low cost.***	High energy consumption due to the drying step. ***Low sustainability.***	[119]
Nanocoating	Direct coating	Enhance and extend the range of functional performance properties such as water-proof tarpaulins, coverings, tents, and upholstery, thus covering application areas such as car seats, food packaging, medical applications. ***Versatile methods***.	Need of modern machinery, impacting the productivity, production speed, and versatility of coating methods. Depends on the adherence of the applied NM to the textile surface. May require additives, therefore ***lowering its sustainability***.	[102]
	Dip coating			
	Spray coating			
	Foam coating			
	Transfer coating			
	Calender coating			
	Hot-melt extrusion coating			
Nanoprinting	Gravure printing	Allows the application of multi-layers of coating. Better distribution and control of deposition on the fabric, thus allowing the creation of versatile patterns. Low-cost and versatile technique with no loss of the printing solution. ***Versatile methods***.	Poor fixation, requiring the use of other chemical agents to improve its washability. ***Low sustainability.***	[107, 115, 119]
	Offset printing			
	Micro/nano printing			
	Pad printing			
	Roll-to-roll printing			
	Screen printing			
	Inkjet printing			
	Slot-die printing			
	Lithography			

Textile substrates commonly used include cotton, wool, polyamide, polypropylene, and polyester. Each substrate, in combination with its corresponding manufacturing process, has a unique sustainability profile that is beyond the scope of this review. However, Zhang et al. (2023) [120] conducted a Life Cycle Assessment (LCA) on wet processing for various textiles (woven polyester, woven cotton, knitted polyester, and knitted cotton), finding that the dyeing unit—driven primarily by electricity consumption and detergent usage—was the largest contributor to environmental impacts, with woven polyester exhibiting the highest and knitted cotton the lowest impacts [120]. Similarly, Hicks et al. [121] noted that for polyester garments, impacts arise mainly from knitting and spinning, whereas for cotton, agricultural production and processing are dominant. Overall, LCAs suggest that cotton-based textiles often incur greater environmental burdens (eutrophication and ecotoxicity) compared to industrial polyester [20]. The ongoing development of AgNM-functionalized textiles increasingly emphasizes the reduction of human health risks [101]. The choice of application methods, solvent recovery practices, textile matrix type, and energy consumption significantly influence sustainability and occupational safety [GC Principles #4, #5, #6, and #12] [73, 107, 115, 117].

ii. Human safety dimension

Similar to the synthesis phase, human safety risks during manufacturing depend largely on the specific technology employed for textile functionalization. The primary exposure routes remain inhalation, dermal contact, and ingestion [70]:

- **Inhalation**: AgNMs can become aerosolized during production, leading to their deposition in the respiratory system. This exposure may cause inflammation, oxidative stress, and lung damage, resulting in reduced lung function [71, 122].
- **Dermal Exposure**: Direct skin contact with AgNMs or contaminated textiles can result in irritation and inflammation. Penetration through the skin depends on the NM size, shape, and surface chemistry [123–125].
- **Ingestion**: Accidental ingestion through hand-to-mouth contact can lead to gastrointestinal absorption and subsequent systemic distribution, potentially accumulating in organs such as the liver and kidneys. Although systemic effects from oral exposure appear limited, further study is required to elucidate NM kinetics [126].

Bottom-up approaches (e.g., spinning techniques) require the dispersion of AgNMs in polymer solutions—often involving hazardous organic solvents and strong acids or bases. Consequently, toxic wastes are generated thus raising challenges for occupational safety [GC Principles #3, #5, #12] [127]. For instance, melt spinning operates at high temperatures, potentially releasing aerosolized dyes, flame retardants, or other hazardous additives, thereby posing increased inhalation risks [128, 129]. In contrast, electrospinning relies solely on a high-voltage field, thereby reducing chemical releases and enhancing inherent process safety [130].

Top-down approaches present distinct safety profiles. In-situ methods mitigate direct exposure to pre-synthesized AgNMs; however, they may involve hazardous precursors. For instance, in-situ synthesis frequently employs nonpolar solvents and toxic reagents that generate hazardous by-products [103]. Montes-Hernandez et al. [131] and Tang et al. [132] demonstrated in-situ methods utilizing NaBH_4_—a reagent with acute toxicity, corrosiveness, and also produces flammable gases when combined with water [133]. Tang et al. [132] additionally reported the use of hydrogen peroxide, a compound that may lead to severe consequences in industrial contexts [134]. The silver source can also be problematic; for example, El-Naggar et al. [135] used silver carbamate, associated with skin irritation and eye damage [136]. These risks are well characterized, facilitating the implementation of appropriate personal protective equipment (PPE) and risk management strategies.

Ex-situ methods primarily expose workers to airborne AgNMs. Occupational exposure limits (OELs) have been established by various organizations: the American Conference of Governmental Industrial Hygienists (ACGIH) sets thresholds at 0.1 mg/m^3^ for silver dust and 0.01 mg/m^3^ for silver soluble compounds [137]; the EU’s Scientific Committee on Occupational Exposure Limits (SCOEL) recommends 0.2–0.5 mg/m^3^ for inhalable silver dust^1^; and the National Institute for Occupational Safety and Health (NIOSH) proposed an OEL of 0.9 g/m^3^ over an 8-hour period for nanosilver [138]. More than only mentioning limits and thresholds, studies as those of Shekftik et al., McCormick et al., West, et al., Trabucco, et al., and Koivisto, et al., have expanded the understanding of NM occupational exposure and associated monitoring methods [139–143].

Shekaftik et al., examining employees at 52 nanotechnology companies, found increased incidence of respiratory, ocular, and cutaneous symptoms (cough, burning throat, eye irritation, skin itching, redness) among workers exposed to NMs. Neurological symptoms, including headaches and sleep disturbances, were notably elevated (over 30%) compared to non-exposed groups. The authors attributed these findings to NM size and high dustiness, facilitating dispersal via minimal movements and airflow. Similarly to the claims made by Shekaftik et al., other studies confirmed the presence of NMs in the air, after conducting an air sampling [141, 144–146]. However, challenges remain, particularly regarding exposure measurement techniques, prompting the use of real-time (online) and offline analytical methods [147]. McCormicks’s et al. highlighted that combining both methods—a multi-metric approach—provides robust characterization of workplace NM exposure. Specifically, for AgNMs, they recommended the NIOSH Manual of Analytical Methods (NMAM) 7303 (ICP-AES) for airborne silver mass and NMAM 9102 (ICP-AES) for surface contamination, supplemented by offline microscopy analyses [140]. West et al. assessed exposure to AgNM additives in spray paint within construction contexts, employing direct-reading instruments and NIOSH Method 7300 combined with electron microscopy. Their findings indicated occupational exposure levels below NIOSH recommendations (< 0.50 µg/m^3^ over an 8-hour period) [143]. Similarly, Koivisto et al. using Near-Field (NF) and Far-Field (FF) assessments combined with mechanistic modeling, reported controlled worker exposure levels (≤ 1.9 µg/m^3^ under worst-case conditions) [139]. Trabucco et al. further demonstrated that using multiple instruments for high-resolution time-based measurements (Optical Particle Counter and Aerodynamic Particle Sizer) enhances reliability in monitoring airborne NM exposures, specifically for hydroxymethylcellulose-coated AgNMs [142]. Although occupational exposure remains a significant concern with expanding nanotechnology applications, these studies collectively indicate manageable exposure risks. They underline the importance of real-time analytical methods and comprehensive monitoring strategies to prevent risky exposures proactively [GC Principles #1, #11].

iii. Environmental dimension

Unlike the synthesis stage, the manufacturing phase benefits from established analytical tools for environmental evaluation. Risk assessment (RA), life cycle assessment (LCA), and socio-economic analysis (SEA) have been widely used to assess the sustainability of emerging technologies from early development through full life cycle integration [24]. Among these, LCA is valued for its ability to quantify a product’s environmental impact measured in terms of CO_2_ emissions, energy consumption, and other standardized metrics (Tukker, 2000). For instance, Pourzahedi et al. [148] conducted a cradle-to-gate LCA on 15 AgNM-enabled products (including baby blankets, t-shirts, towels, and socks) to determine the contribution of AgNMs to overall impacts. While chemical reduction using sodium borohydride was generally advantageous, techniques like flame-spray pyrolysis exhibited the highest environmental burdens. Depending on product composition and silver loading, the environmental impacts ranged widely from 1% to 99%, with the majority attributable to the polymeric matrix rather than AgNMs themselves, underscoring the importance of considering atom economy and efficient use of materials in textile manufacturing [GC Principle #2].

Sarker and Bartok reported an increasing interest in green textile manufacturing techniques. For instance, 62% of research papers on the subject have been released in the last five years, accounting 90% of them were exclusively research articles [149]. The textile and apparel industries use a variety of sustainable production techniques, including energy-efficient manufacturing techniques, water conservation techniques, waste reduction and recycling strategies, and chemical management and replacement [GC Principles #3, #4, #5, and #6] [150]. For instance, natural fibres such as cotton, wool, and silk, generally have a lower carbon footprint (CF) when compared to synthetic fabrics such as polyester and nylon, requiring more energy and chemicals during production [151]. Therefore, lowering its environmental impact entails a shift toward employing natural fibres, such as organic cotton, wool, and hemp, renowned for their eco-friendly cultivation practices. In addition, fabrics manufactured through eco-friendly methods, such as closed-loop systems and sustainable production processes, have a significantly lower CF than those produced using conventional means [151]. Conversely, Abu-Qdais et al. [20] compared four different AgNM synthesis methods and evaluated four AgNM-containing textile products via LCA. Even when assessing “green” synthesis routes, their analysis consistently indicated that the manufacturing stage accounted for the greatest environmental impacts across the life cycle. Differences between studies likely stem from methodological variations, such as system boundaries; Abu-Qdais et al. excluded silver extraction and fabric production, whereas Pourzahedi et al. included them, attributing significant impacts to mining and electricity consumption [20, 148, 152]. Such discrepancies highlight the necessity for standardized and comprehensive assessment methods, enabling pollution prevention and the design of inherently safer textile processes [GC Principles #11 and #12].

The textile and fashion industries must continue to put a high priority on sustainable manufacturing techniques in the future. Businesses must adopt more environmentally friendly production methods, materials, and supply chain management techniques, as well as integrate eco-design ideas into their product development processes [GC Principles #3, #4, and #12] [150]. Nonetheless, the studies concur that the use and end-of-life phases pose the greatest ecotoxicological risks. Integrating LCA within the SSbD framework is especially beneficial, as it requires a holistic evaluation of both NM production processes and the complete product life cycle, including textile manufacturing. It remains an open question whether a unified LCA model or multiple integrated assessments best captures the total environmental impact.

c. Use phase

Throughout a product’s use phase, the potential release of NMs from NEPs poses concerns for both public health and the environment. During routine use and care, such as washing, this NMs may be released into various matrices, including air, sweat, and effluent water [153], potentially leading to adverse effects. Given the importance of understanding NM behaviour and its impacts, comprehensive characterization is essential to inform risk mitigation [101]. Part of what needs to be evaluated is the NM release, fate, leaching, and toxicity.

i. Human safety dimension

NEPs exhibit a wide range of silver concentrations from 0.9 to 1358.3 µg Ag/g in socks, 0.99–15.1 µg Ag/g in antibacterial fabrics, and 30–270 µg Ag/g in various household textiles [20]. Studies indicate considerable variability in NMs released from textiles ranges from less than 1% to nearly 100%, depending on the content of NMs contained within the textiles [153]. The migration of AgNMs into human skin is of particular concern, given uncertainties regarding their toxicity [154]. Recent research has indicated the potential permeation of metallic NMs into the skin, raising concerns about human health [153]. Consequently, precise quantification of AgNM migration is critical for ensuring public safety and regulatory agencies closely monitor these levels [73].

For example, Wagener et al. [155] concluded that wearing AgNM-treated textiles can expose consumers to dissolved Ag ions highlighting the importance of designing safer chemicals and textile treatments [GC Principle #4]. Similarly, Gagnon et al. [156] reported that weathering of textiles leads to AgNM release via degradation of non-nano coatings, resulting in the formation of various silver forms, including spherical NMs and nanoplates. Patch et al. [157] proposed a novel approach to analyse AgNM release from conventional textiles. Their simulation of human weathering using artificial sweat revealed a release of 67 ± 11 mg Ag/kg of textile, with the silver present in ionic, metallic, and chlorinated forms, as well as nanosheets and particulates. Peloquin et al. [158] explored how artificial sweat conditions (e.g., pH, chloride concentration) influence AgNM physicochemical transformations, aggregation, and stability across particle sizes (20–100 nm). Their results indicated that lower pH and higher chloride concentrations increased the hydrodynamic diameter, and that 75 nm particles were the most stable. Complementary analyses (UV-Vis, spICP-MS, DLS) suggested aggregation, particularly for 50 and 100 nm particles [158].

Multiple studies have investigated the release of AgNMs into various media such as artificial sweat and saliva, demonstrating that chemical weathering significantly influences the quantity, speciation, morphology, and stability of released silver species [159–161]. Beigzadeh et al. (2024) observed no detectable NMs release from 57% of examined textiles under artificial sweat conditions, suggesting these textiles are designed to minimize dermal transfer, thus aligning with safer design principles [GC Principles #4, #10] [153]. Aging also plays a critical role in AgNM release, with experimental conditions such as light exposure, temperature, and humidity influencing the outcomes; however, gaps remain in the methodologies used to evaluate these effects. In this context [157], developed a low-resource method to simulate human weathering of textiles, revealing that AgNM-enabled textiles can release silver following wear before laundering [162]. conducted in vitro experiments to assess the dermal penetration of AgNMs in human and pig skin. Their results indicated that coated AgNMs exhibit minimal dermal penetration, with less than 10% of the applied Ag dose being absorbed. Most of the absorbed silver localized in the stratum corneum with only trace amounts reaching the deeper dermal layer [154]. Additional studies further revealed significantly higher AgNM penetration rates through damaged skin compared to intact skin. For example, after 24 h, median penetration through injured skin (2.32 ng/cm^2^) was approximately five times higher than intact skin (0.46 ng/cm^2^) [125, 154, 163, 164]. These results emphasize that both NM physicochemical properties and the integrity of the skin barrier play critical roles in toxicity outcomes. Consequently, tailoring AgNM surface chemistry and functionalization can enhance dermal safety, supporting inherently safer design approaches in textile applications [GC Principles #4].

ii. Environmental dimension

The use phase of textiles presents significant environmental impacts, predominantly associated with laundering practices. Laundering alone contributes up to 15 kg CO_2_-equivalent emissions per shirt over its lifetime, while textile production further contributes to ozone depletion (1 × 10^−7^ kg CFC-11 eq) and carcinogenic potential (1.8 × 10^−7^ CTUh per shirt lifetime) [121]. Energy-intensive washing and drying processes increase environmental burdens and facilitate the release of AgNM into the environment [20]. NEPs’ manufacturers assert that their products require less laundering than conventional options due to odour reduction, yet no data has been published so far confirming change in consumer laundering behaviour.

Studies by Radwan & Eljamal, Shilo Nesa Sherlin et al., and Moloi, et al. highlight AgNM release into wash water from functionalized textiles [113, 165, 166]. Limpiteeprakan et al. and Shilo Nesa Sherlin et al. observed that the pad-dry-cure method yielded limited durability in NM fixation during the initial wash cycles, due to primarily superficial binding [113, 161]. To enhance washing durability and minimize AgNM release, researchers have explored various pre- and post-treatment strategies consistent with designing safer textiles [GC Principle #4]. Pre-treatments using molecular adhesives like bovine serum albumin (BSA) improve NM binding, while post-treatment embedding techniques (e.g., polydimethylsiloxane [PDMS], polyurethane [PU] sealing, screen-printed PU layers, sonochemical deposition, enzyme crosslinking) effectively reduce leaching [113, 167]. Moreover, fabric composition significantly affects AgNM retention: a 35:65 cotton-polyester blend retained over half (52%) of its original NM content after 20 washes, suggesting blending may enhance durability [GC Principles #4, #10] [19]. Similarly, natural fibers such as wool show superior NMs adhesion (10 mg/g) compared untreated cotton (2.3 mg/g), bleached cotton (>1 mg/g), polyamide (>0.62 mg/g), and polyester (>0.28 mg/g), emphasizing the advantage of renewable feedstocks and substrates in reducing environmental release [GC Principles #7, #10] [20, 131]. Despite these advances, few studies have systematically assessed the influence of variables such as detergent type, temperature, and fabric composition on AgNM release. For instance, a dataset compiled by Mirzaei et al. [168] detailed factors such as washing machine type, detergent, wash cycles, fabric type, and retained antimicrobial efficiency, yet lacked quantitative release data. Further research is thus required to elucidate the factors governing AgNM release and to develop strategies that mitigate NM leaching.

Innovative strategies currently explored to reduce NM release and enhance functionalization durability include:

1. **Porous materials** Zeolites show potential for controlling particle size and uniform distribution; though research into Ag-zeolite release during washing remains limited [73, 169].
2. **Polysaccharide matrices** Biodegradable materials such as chitosan, alginate, starch, cyclodextrins, and cellulose can stabilize AgNMs and reduce leaching, aligning closely with green chemistry’s principles [GC Principles #7, #10] [170].
3. **Polymeric matrices** Textiles incorporating conjugated polymer NMs have demonstrated robust durability and multifunctionality and reduced environmental release, suitable for diverse sectors including healthcare, sports, fashion, and defence [171].
4. **Nanotubes encapsulation** Tubular nanostructures allow for the encapsulation of AgNMs, as evidenced by the integration of AgNMs into TiO_2_ nanotube arrays and AgNM-halloysite nanocomposites, which enhance durability and offer promising biomedical applications [73].

Efforts to “green” these techniques increasingly focus on substituting hazardous chemicals with safer alternatives, such as sugars as reducing agents, polyethylene glycol as stabilizers, or biological agents derived from bacteria, fungi, algae, or plants. The incorporation of auxiliary supports such as polysaccharides, zeolites, polymer matrices, and nanotubes and additional post-treatment methods like UV or thermal curing further exemplify strategies designed to enhance the safety, durability, and environmental sustainability of AgNM-enabled textiles [GC Principles #3, #4, #5, #7, and #12] [67, 116, 170, 172, 173]. Ultimately, the interplay between textile substrate characteristics, AgNM physicochemical properties, functionalization parameters, and subsequent environmental, occupational, and consumer safety impacts must be assessed on a case-by-case basis.

d. End of life
  i. Environmental dimension

Textiles account roughly 5% of municipal waste annually [174]. In the European Union, most end-of-life textiles are primarily exported for reuse or disposed of via conventional landfilling and incineration, highlighting a missed opportunity for sustainable waste management [GC Principle #1] [175]. For AgNM-enabled textiles, the NMs are considered waste once they detach from the fabric. Consequently, the predominant environmental exposure route during the use phase is through wastewater generated during washing cycles [153, 165, 166].

**Fig. 2:**
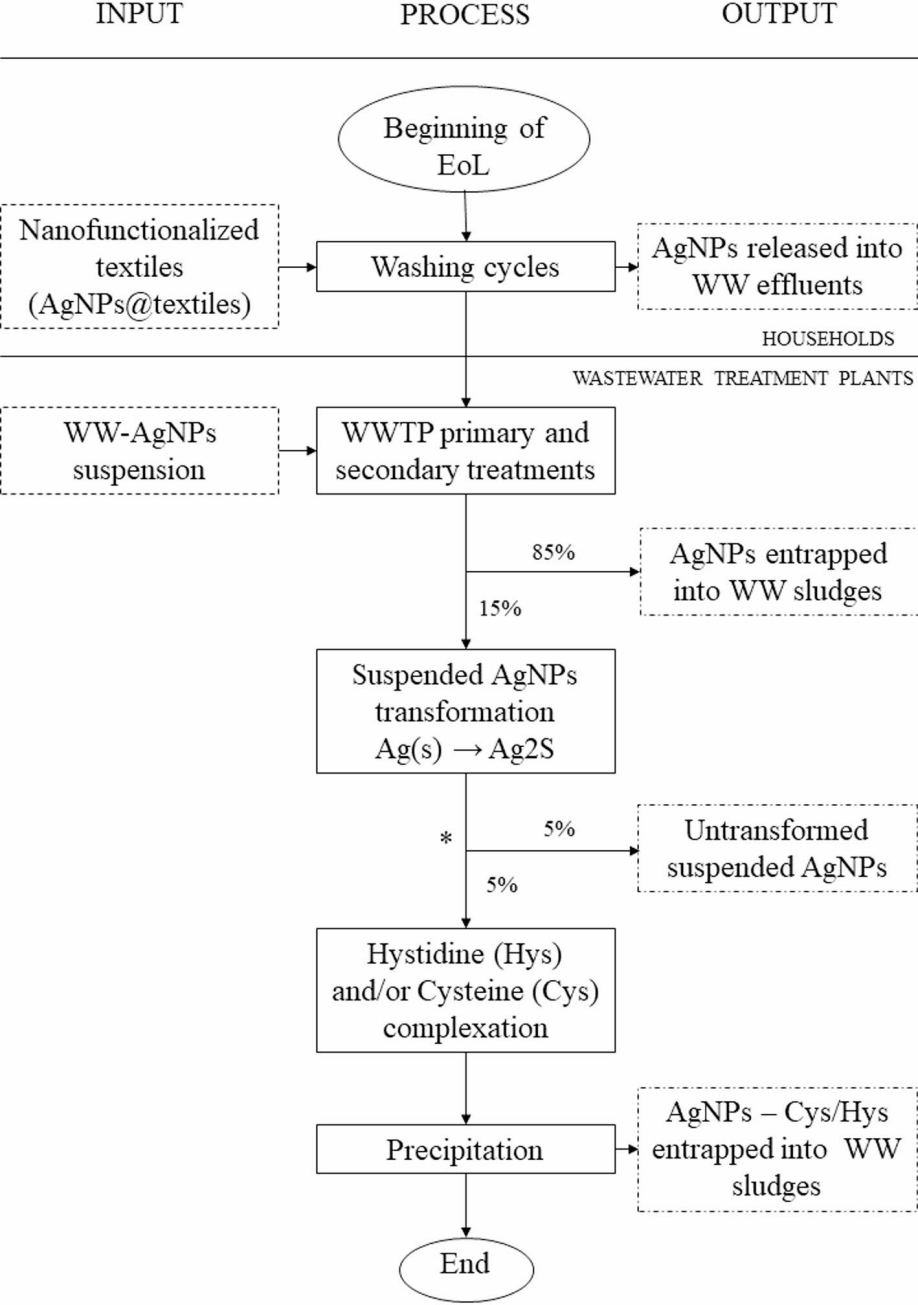
AgNMs EoL flux and fate through the WWTP processes. *The 5% missing was not found to be reported

When in their free form—detached from matrices—AgNMs pose significant risks to microbial communities essential for wastewater treatment processes AgNMs can inhibit the growth of bacteria and other microorganisms essential for effective wastewater treatment [176] and adversely affect **aquatic and terrestrial microbial** populations that underpin many ecosystems [177]. Toxicity studies indicate that AgNMs reveal substantial ecotoxicity of AgNMs, with median L(E)C_50_ values indicating very high toxicity towards crustaceans (0.018 mg L^−1^) and algae (0.195 mg L^−1^), high toxicity towards fish (1.8 mg L^−1^), and moderate toxicity to protozoa (16 mg L^−1^). These LC_50_, EC_50_, and IC_50_ values are critical for guiding improvements in the physicochemical properties (e.g., size and shape) of AgNMs and for developing manufacturing practices that mitigate environmental impacts [GC Principles #4, #10] [178]. Complementing this, Bonfanti et al. found that NMs’ morphology and zeta potential significantly influence toxicity toward zebrafish embryos, with coating agents playing a critical role in enhancing or mitigating toxicity [179]. Similarly, Moloi et al. [165] identified high ecological risks to fish resulting from AgNM release from textiles. AgNM release during laundering raises environmental concerns due to NMs transport into aquatic ecosystems [153, 166]. Wastewater treatment plants (WWTPs), however, effectively capture around 85% of AgNMs in sewage sludge following preliminary and secondary treatments, with only approximately 5% remaining suspended and another 5% converted into silver sulfide (Ag_2_S) (see Fig. 2) [180, 181]. Notably, AgNMs do not agglomerate into larger clusters during treatment, allowing them to preserve their nanoscale properties. Transformations within sludge typically yield secondary Ag_2_S particles through complexation reactions with molecules such as histidine or cysteine, with extractable Ag concentrations as low as 0.008–1.7% [180, 182]. Long-term environmental studies indicate minimal Ag extraction from soils (~ 2.9 µg/kg after 400 days) and negligible plant uptake, suggesting low phytotoxic potential [182].

AgNMs are expected to be retained in sewage sludge in WWTPs [166, 183, 184]. Due to regulatory restrictions on dumping sewage sludge at sea in both Europe (EU Directive 91/271/EEC; EEC, 1991) and the United States (Ocean Dumping Ban Act of 1998), sludge from WWTPs is often repurposed as fertilizer for agricultural soils. However, this practice may facilitate the transfer of AgNMs to aquatic systems via surface runoff [19]. Sludge destined for landfills also poses a leaching risk, potentially releasing AgNMs into nearby water bodies [185, 186]. While recovery or recycling of AgNMs from sludge is theoretically feasible, complex chemical transformations currently limit practical implementation, highlighting the necessity of designing NMs and functionalized textiles for easier recovery or safer degradation at their end-of-life [GC Principles #1, #10].

Notably, the strong affinity between Ag-derived compounds and sludge can be advantageous if disposal is the only viable option. Currently, landfilling is the predominant disposal method for WWTP sludge [20, 187]. Nevertheless, the mobility of Ag in the sludge–soil system remains a concern. Despite the high stability and negligible ecotoxicity of Ag_2_S in sludge [182], LCA studies of AgNM-functionalized textiles report that the disposal phase has the highest ecotoxicological impact [20, 148]. It is important to note, however, that many LCA models do not account for AgNM entrapment during wastewater treatment, underscoring the need for clearly defined system boundaries and recognizing the pivotal role of WWTPs in the SSbD framework [GC Principles #1, #11]. Wang et al. [182] reported negligible extraction of silver from sludge, unaffected by variations in pH and temperature. Only high chloride concentrations slightly increased Ag extractability, but concentrations remained negligible. Additionally, plant uptake of silver from sludge-amended soils proved minimal and non-phytotoxic, further supporting the relative environmental stability of Ag_2_S in disposal contexts [GC Principle #10] [182]. At their end-of-life, textiles typically face disposal due to limited recyclability [188]. The fate of AgNM-enabled textiles during the end-of-life phase has received relatively little attention, with only a few studies linking this phase to earlier life cycle stages [20, 189, 190]. Disposal scenarios generally include incineration followed by landfilling or direct landfilling. Current literature suggests that, under municipal sewage treatment conditions—where AgNMs are largely sequestered in sludge—the environmental risk from AgNMs is minimal. However, this conclusion is based on a linear economy model rather than a circular one. In light of policies such as the European Green Deal and the Chemical Strategy for Sustainability (CSS), transitioning to a circular economy for AgNMs is imperative. [GC Principles #1, #7] This transition would benefit from the development of methods to retrieve residual Ag-containing compounds from sludge, a challenge that to our knowledge, remains unaddressed in the literature. In accordance with the EU Waste Framework Directive [191] and the SSbD framework, end-of-life protocols must prioritize recycling or reuse; when recycling is unfeasible, products must be designed to degrade into innocuous components [GC Principles #1, #10] [82]. Nonetheless, each textile disposal method—reuse, recycling, incineration, or landfilling—collectively contributes to the product’s cumulative energy demand and environmental impact, emphasizing the critical role of energy-efficient and sustainably designed end-of-life treatments [GC Principle #6] [192].

## Limitations

This review provides a structured, life cycle-oriented synthesis of AgNMs in textiles, aligned with Green Chemistry and emerging sustainability frameworks. Nonetheless, several limitations should be acknowledged regarding both the underlying literature and the review process itself.

First, the analysis remains largely qualitative due to the heterogeneity of available studies and the lack of standardized methodologies. Differences in scope, terminology, and experimental protocols particularly regarding AgNM release, transformation, and toxicity, limit the extent to which results can be directly compared. No formal meta-analysis was conducted, as variability in design and reporting precludes consistent statistical aggregation. In addition, although some green synthesis strategies are relatively well characterized, the overall literature is heavily weighted toward the early stages of the life cycle. The manufacturing, use, and end-of-life phases remain comparatively underexplored, with existing data often limited to case-specific or descriptive accounts. For instance, long-term leaching behaviour, transformation pathways in complex environments, and the fate of AgNMs in wastewater or soils are insufficiently understood.

There are also notable gaps in the technical and operational dimensions of AgNM integration. While many green synthesis routes demonstrate promise at the laboratory scale, their scalability, process stability, and cost-efficiency under industrial conditions remain unclear. Similarly, integration methods—such as surface coating, embedding, or fiber spinning—are inconsistently reported and rarely assessed for long-term performance or sustainability trade-offs. Furthermore, sustainability metrics specific to nanomaterial-enabled textile production are still lacking in both academic and industrial practice. From a regulatory and systemic perspective, the review reflects a European policy emphasis, including references to REACH and the SSbD framework. Although these initiatives are influential, their global applicability may be limited by differences in regulatory infrastructure, market maturity, and enforcement capacity.

To address these systemic limitations and ensure long-term viability, regulatory systems must evolve alongside these innovations. Although SSbD is gaining prominence in nanotechnology governance [193–195], fragmented awareness and inconsistent oversight continue to hinder its widespread adoption. Numerous frameworks and tools—including GUIDEnano, the GRACIOUS^2^ framework, the ASINA-Expert System^3^, and the Nano-Risk Governance Portal^4^—are now supporting efforts to institutionalize SSbD [193, 196]. In parallel, initiatives like the EU NanoSafety Cluster^5^ and metrics such as Physical-Chemical Features (PCFs), Key Decision Factors (KDFs), and Key Performance Indicators (KPIs) offer actionable methodologies for implementation [197].

Finally, this review prioritizes technical, environmental, and policy dimensions of AgNMs, and does not address in depth the socioeconomic, market, or ethical considerations surrounding nanotechnology adoption in the textile sector. Future research may benefit from more integrative approaches that combine technical evaluation with stakeholder perspectives and systemic sustainability assessments, as well as addressing cost implications of transitioning to SSbD-compliant technologies.

## Conclusion and future perspectives

The integration of AgNMs into textiles presents a compelling intersection of innovation and sustainability challenges. While their functional advantages are well-recognized, their environmental and health implications remain insufficiently addressed. The SSbD framework emerges as a pivotal strategy, guiding material innovation toward holistic responsibility from the outset.

This review underscores the need to embed SSbD principles across all stages of the AgNM-functionalized textile lifecycle—from greener synthesis methods to responsible end-of-life strategies. Advances such as bio-based synthesis and encapsulation technologies show promise, but significant variability in real-world conditions and limited recyclability continue to hamper progress. The mismatch between technological advancement and regulatory maturity highlights a critical governance gap.

Going forward, interdisciplinary collaboration will be essential to identify actionable leverage points along the value chain. More importantly, fostering stakeholder alignment—across researchers, industry, regulators, and consumers—is vital for the successful implementation of SSbD. By adopting a lifecycle-thinking approach rooted in the Green Chemistry Principles, the field can move toward a future where nanotechnology contributes meaningfully to innovation without compromising sustainability.

## Data Availability

No datasets were generated or analysed during the current study.

## Footnotes

1 Recommendations of the SCOEL - ECHA.

2 Draft GRACIOUS Guidance_final version.pdf.

3 ASINA project | Safe-by-design of Nano Product Development.

4 Nano-Risk Governance Portal.

5 NSC – Network for Safety and sustainability of Chemicals and materials.

## References

[1] Krifa M, Prichard C. Nanotechnology in textile and apparel research: an overview of technologies and processes. J Text Inst. 2020;111(12):1778–93.

[2] Ali F, Neha K, Sharma K, Khasimbi S, Chauhan G. Nanotechnology-based medicinal products and patents: a promising way to treat psoriasis. Curr Drug Deliv. 2022;19(5):587–99. 35081890 10.2174/1567201819666220126163943

[3] Nie P, Zhao Y, Xu H. Synthesis, applications, toxicity and toxicity mechanisms of silver nanoparticles: a review. Ecotoxicol Environ Saf. 2023;253:114636. 36806822 10.1016/j.ecoenv.2023.114636

[4] Santos AC, Morais F, Simões A, Pereira I, Sequeira JAD, Pereira-Silva M, Veiga F, Ribeiro A. Nanotechnology for the development of new cosmetic formulations. Expert Opin Drug Deliv. 2019;16(4):313–30. 30793641 10.1080/17425247.2019.1585426

[5] Sharma C, Dhiman R, Rokana N, Panwar H, Nanotechnology. An untapped resource for food packaging. Front Microbiol [Internet]. 2017 Sep 12 [cited 2025 Jan 28];8. https://www.frontiersin.org/journals/microbiology/articles/10.3389/fmicb.2017.01735/full 10.3389/fmicb.2017.01735 PMC5601076 28955314

[6] Shi Q, He T, Lee C. More than energy harvesting: combining triboelectric nanogenerator and flexible electronics technology for enabling novel micro-/nano-systems. Nano Energy. 2019;57:851–71.

[7] Zhao L, Lu L, Wang A, Zhang H, Huang M, Wu H, Xing B, Wang Z, Ji R. Nano-biotechnology in agriculture: use of nanomaterials to promote plant growth and stress tolerance. J Agric Food Chem. 2020;68(7):1935–47. 32003987 10.1021/acs.jafc.9b06615

[8] Dhanasekaran R, Sreenatha Reddy S, Girish Kumar B, Anirudh AS. Shape memory materials for bio-medical and aerospace applications. Mater Today Proc. 2018;5(10, Part 1):21427–35.

[9] Eyvaz M, Arslan S, Gürbulak E, Yüksel E, Eyvaz M, Arslan S, Gürbulak E, Yüksel E. Textile materials in liquid filtration practices: current status and perspectives in water and wastewater treatment. In: Textiles for advanced applications [Internet]. IntechOpen; 2017 [cited 2025 Jan 30]. https://www.intechopen.com/chapters/55837

[10] Ismar E, Kurşun Bahadir S, Kalaoglu F, Koncar V. Futuristic clothes: electronic textiles and wearable technologies. Glob Chall. 2020;4(7):1900092. 32642074 10.1002/gch2.201900092 PMC7330505

[11] Shah MA, Pirzada BM, Price G, Shibiru AL, Qurashi A. Applications of nanotechnology in smart textile industry: a critical review. J Adv Res. 2022;38:55–75. 35572402 10.1016/j.jare.2022.01.008 PMC9091772

[12] EUON EUO for N. Market - EUON [Internet]. 2021 [cited 2025 Mar 25]. https://euon.echa.europa.eu/nanodata/sectors/manufacturing/overview/market

[13] The Business Research Company. Nanotechnology clothing market report 2025 - nanotechnology clothing market share and analysis [Internet]. 2025 [cited 2025 Jan 28]. https://www.thebusinessresearchcompany.com/report/nanotechnology-clothing-global-market-report

[14] SCENIHR SC on E and NIHR. Nanosilver: safety, health and environmental effects and role in antimicrobial resistance [Internet]. Nanosilver: safety, health and environmental effects and role in antimicrobial resistance. 2014 [cited 2025 Mar 25]. https://health.ec.europa.eu/publications/nanosilver-safety-health-and-environmental-effects-and-role-antimicrobial-resistance_en

[15] SCENIHR SC on E and NIHR. Final opinion on nanosilver: safety, health and environmental effects and role in antimicrobial resistance [Internet]. Final opinion on Nanosilver: safety, health and environmental effects and role in antimicrobial resistance. 2014 [cited 2025 Mar 25]. https://health.ec.europa.eu/other-pages/health-sc-basic-page/final-opinion-1_en

[16] SCCS SC on CS. Colloidal Silver (nano) [Internet]. Colloidal Silver (nano). 2018 [cited 2025 Mar 25]. https://health.ec.europa.eu/publications/colloidal-silver-nano_en

[17] The Nanodatabase. Nano-silver is no longer approved under the Biocidal Products Regulation (BPR) for specific uses [Internet]. 2021 [cited 2025 Mar 25]. https://nanodb.dk/en/news/nano-silver-is-no-longer-approved-under-the-biocidal-products-regulation-bpr-for-specific-uses/

[18] European Commission. Commission Regulation (EU) 2024/858 of 14 March 2024 amending Regulation (EC) No 1223/2009 of the European Parliament and of the Council as regards the use of the nanomaterials Styrene/Acrylates copolymer, Sodium Styrene/Acrylates copolymer, Copper, Colloidal Copper, Hydroxyapatite, Gold, Colloidal Gold, Gold Thioethylamino Hyaluronic Acid, Acetyl heptapeptide-9 Colloidal gold, Platinum, Colloidal Platinum, Acetyl tetrapeptide-17 Colloidal Platinum and Colloidal Silver in cosmetics products [Internet]. 2024 [cited 2025 Mar 25]. https://eur-lex.europa.eu/eli/reg/2024/858/oj/eng

[19] McGillicuddy E, Murray I, Kavanagh S, Morrison L, Fogarty A, Cormican M, Dockery P, Prendergast M, Rowan N, Morris D. Silver nanoparticles in the environment: sources, detection and ecotoxicology. Sci Total Environ. 2017;575:231–46. 27744152 10.1016/j.scitotenv.2016.10.041

[20] Abu-Qdais HA, Abu-Dalo MA, Hajeer YY. Impacts of nanosilver-based textile products using a life cycle assessment. Sustainability. 2021;13(6):3436.

[21] Aguda ON, Lateef A. Recent advances in functionalization of nanotextiles: a strategy to combat harmful microorganisms and emerging pathogens in the 21st century. Heliyon [Internet]. 2022 Jun 1 [cited 2025 Jan 28];8(6). https://www.cell.com/heliyon/abstract/S2405-8440(22)01049-0 10.1016/j.heliyon.2022.e09761 PMC9249839 35789866

[22] Abbate E, Garmendia AI, Bracalente G, Mancini L, Tosches D, Rasmussen K, Bennett MJ, Rauscher H, Sala S. Safe and sustainable by design chemicals and materials - methodological guidance [Internet]. JRC Publications Repository. 2024 [cited 2025 Mar 4]. https://publications.jrc.ec.europa.eu/repository/handle/JRC138035

[23] Guinée JB, Heijungs R, Vijver G, Peijnenburg MM, Mendez WJG. The meaning of life … cycles: lessons from and for safe by design studies. Green Chem. 2022;24(20):7787–800.

[24] Salieri B, Barruetabeña L, Rodríguez-Llopis I, Jacobsen NR, Manier N, Trouiller B, Chapon V, Hadrup N, Jiménez AS, Micheletti C, Merino BS, Brignon JM, Bouillard J, Hischier R. Integrative approach in a safe by design context combining risk, life cycle and socio-economic assessment for safer and sustainable nanomaterials. NanoImpact. 2021;23:100335. 35559836 10.1016/j.impact.2021.100335

[25] Soeteman-Hernandez LG, Apostolova MD, Bekker C, Dekkers S, Grafström RC, Groenewold M, Handzhiyski Y, Herbeck-Engel P, Hoehener K, Karagkiozaki V, Kelly S, Kraegeloh A, Logothetidis S, Micheletti C, Nymark P, Oomen A, Oosterwijk T, Rodríguez-LLopis I, Sabella S, Sanchez Jiménez A, Sips AJAM, Suarez- Merino B, Tavernaro I, van Engelen J, Wijnhoven SWP, Noorlander CW. Safe innovation approach: towards an agile system for dealing with innovations. Mater Today Commun. 2019;20:100548.

[26] Brunelli A, Serrano-Lotina A, Bañares A, Alcolea-Rodriguez M, Blosi V, Costa M, Ortelli A, Peijnenburg S, Fito W, Fernandez CG, Hermosilla ES, Soeteman-Hernández JG, Aguirre L, Rauscher IG, Murphy H, Stone F, Balbuena V, Cormano JL, Pizzol JM, Hristozov L, Marcomini D, Badetti A. Safe-by-design assessment of an SiO_2_@ZnO multi-component nanomaterial used in construction. Environ Sci Nano. 2025;12(1):762–76.

[27] Cassee FR, Bleeker EAJ, Durand C, Exner T, Falk A, Friedrichs S, Heunisch E, Himly M, Hofer S, Hofstätter N, Hristozov D, Nymark P, Pohl A, Soeteman-Hernández LG, Suarez-Merino B, Valsami-Jones E, Groenewold M. Roadmap towards safe and sustainable advanced and innovative materials. (Outlook for 2024–2030). Comput Struct Biotechnol J. 2024;25:105–26. 38974014 10.1016/j.csbj.2024.05.018 PMC11225617

[28] Furxhi I, Perucca M, Blosi M, Lopez de Ipiña J, Oliveira J, Murphy F, Costa AL. ASINA Project: Towards a Methodological Data-Driven Sustainable and Safe-by-Design Approach for the Development of Nanomaterials. Front Bioeng Biotechnol [Internet]. 2022 Jan 28 [cited 2025 Jan 30];9. https://www.frontiersin.org/journals/bioengineering-and-biotechnology/articles/. 10.3389/fbioe.2021.805096/full 10.3389/fbioe.2021.805096 PMC8832976 35155410

[29] Soeteman-Hernández LG, Tickner A, Dierckx J, Kümmerer A, Apel K, Strömberg C. E. Accelerating the industrial transition with safe-and-sustainable-by-design (SSbD). RSC Sustain [Internet]. 2025 [cited 2025 Mar 24]; https://pubs.rsc.org/en/content/articlelanding/2025/su/d4su00809j

[30] Stoycheva S, Peijnenburg W, Salieri B, Subramanian V, Oomen AG, Pizzol L, Blosi M, Costa A, Doak SH, Stone V, Livieri A, Kestens V, Garmendia I, Rauscher H, Hunt N, Hristozov D, Soeteman-Hernández LG. A Conceptual Framework for Safe-and-Sustainable-by-Design to Support Sustainable Business Model Innovation and New Product Development. Sustain Circ NOW [Internet]. 2025 [cited 2025 Mar 24];02(continuous publication). Available from: http://www.thieme-connect.de/DOI/DOI?10.1055/a-2498-8902

[31] Caldeira C, Farcal R, Garmendia AI, Mancini L, Tosches D, Amelio A, Rasmussen K, Rauscher H, Riego SJ, Sala S. Safe and sustainable by design chemicals and materials: framework for the definition of criteria and evaluation procedure for chemicals and materials [Internet]. JRC Publications Repository. 2022 [cited 2022 Dec 12]. https://publications.jrc.ec.europa.eu/repository/handle/JRC128591

[32] Anastas PT, Warner JC. Principles of green chemistry. In: Anastas PT, Warner JC, editors. Green Chemistry: Theory and Practice [Internet]. Oxford University Press; 2000 [cited 2025 Jan 30]. p. 0. 10.1093/oso/9780198506980.003.0004

[33] Motta G, Gualtieri M, Saibene M, Bengalli R, Brigliadori A, Carrière M, Mantecca P. Preliminary toxicological analysis in a safe-by-design and adverse outcome pathway-driven approach on different silver nanoparticles: assessment of acute responses in A549 cells. Toxics. 2023;11(2):195. 36851069 10.3390/toxics11020195 PMC9965967

[34] Vishwanath R, Negi B. Conventional and green methods of synthesis of silver nanoparticles and their antimicrobial properties. Curr Res Green Sustain Chem. 2021;4:100205.

[35] Rafique M, Sadaf I, Rafique MS, Tahir MB. A review on green synthesis of silver nanoparticles and their applications. Artif Cells Nanomed Biotechnol. 2017;45(7):1272–91. 27825269 10.1080/21691401.2016.1241792

[36] Yusuf M. Silver nanoparticles: synthesis and applications. Handb Ecomater. 2018;2343–56.

[37] Restrepo CV, Villa CC. Synthesis of silver nanoparticles, influence of capping agents, and dependence on size and shape: a review. Environ Nanotechnol Monit Manag. 2021;15:100428.

[38] Pastoriza-Santos I, Liz-Marzán LM. Formation and stabilization of silver nanoparticles through reduction by N,N-dimethylformamide. Langmuir. 1999;15(4):948–51.

[39] Gurusamy V, Krishnamoorthy R, Gopal B, Veeraravagan V. Systematic investigation on hydrazine hydrate assisted reduction of silver nanoparticles and its antibacterial properties. Inorg Nano-Met Chem. 2017;47(5):761–7.

[40] Won HI, Nersisyan H, Won CW, Lee JM, Hwang JS. Preparation of porous silver particles using ammonium formate and its formation mechanism. Chem Eng J. 2010;156(2):459–64.

[41] EPA, Environmental Protection Agency, Dimethylformamide. CASRN 68-12-2 | IRIS | US EPA, ORD [Internet]. 2000 [cited 2025 Feb 19]. Available from: https://iris.epa.gov/ChemicalLanding/&substance_nmbr=511

[42] EPA, Environmental Protection Agency. Hydrazine/Hydrazine sulfate CASRN 302-01-2 | IRIS | US EPA, ORD [Internet]. 2000 [cited 2025 Feb 19]. https://iris.epa.gov/ChemicalLanding/&substance_nmbr=352

[43] Mirzaei A, Janghorban K, Hashemi B, Bonyani M, Leonardi SG, Neri G. Characterization and optical studies of PVP-capped silver nanoparticles. J Nanostruct Chem. 2017;7(1):37–46.

[44] Mohamad Kasim AS, Ariff AB, Mohamad R, Wong FWF. Interrelations of synthesis method, polyethylene glycol coating, physico-chemical characteristics, and antimicrobial activity of silver nanoparticles. Nanomaterials. 2020;10(12):2475. 33321788 10.3390/nano10122475 PMC7764142

[45] Ajitha B, Reddy YAK, Sreedhara Reddy P, Jeon HJ, Won Ahn C. Role of capping agents in controlling silver nanoparticles size, antibacterial activity and potential application as optical hydrogen peroxide sensor. RSC Adv. 2016;6(42):36171–9.

[46] Logaranjan K, Raiza AJ, Gopinath SCB, Chen Y, Pandian K. Shape- and size-controlled synthesis of silver nanoparticles using Aloe Vera plant extract and their antimicrobial activity. Nanoscale Res Lett. 2016;11(1):520. 27885623 10.1186/s11671-016-1725-x PMC5122529

[47] Julinová M, Vaňharová L, Jurča M. Water-soluble polymeric xenobiotics—polyvinyl alcohol and polyvinylpyrrolidon—and potential solutions to environmental issues: a brief review. J Environ Manage. 2018;228:213–22. 30223180 10.1016/j.jenvman.2018.09.010

[48] Pietrelli L, Ferro S, Reverberi AP, Vocciante M. Removal of polyethylene glycols from wastewater: a comparison of different approaches. Chemosphere. 2021;273:129725. 33529796 10.1016/j.chemosphere.2021.129725

[49] Abada E, Mashraqi A, Modafer Y, Al Abboud MA, El-Shabasy A. Review green synthesis of silver nanoparticles by using plant extracts and their antimicrobial activity. Saudi J Biol Sci. 2024;31(1):103877. 38148949 10.1016/j.sjbs.2023.103877 PMC10749906

[50] Roy A, Bulut O, Some S, Kumar Mandal A, Deniz Yilmaz M. Green synthesis of silver nanoparticles: biomolecule-nanoparticle organizations targeting antimicrobial activity. RSC Adv. 2019;9(5):2673–702. 35520490 10.1039/c8ra08982e PMC9059941

[51] Blosi M, Brigliadori A, Ortelli S, Zanoni I, Gardini D, Vineis C, Varesano A, Ballarin B, Perucca M, Costa AL. Re-designing nano-silver technology exploiting one-pot hydroxyethyl cellulose-driven green synthesis. Front Chem [Internet]. 2024 Aug 14 [cited 2025 Feb 27];12. https://www.frontiersin.org/journals/chemistry/articles/10.3389/fchem.2024.1432546/full 10.3389/fchem.2024.1432546 PMC11349673 39206438

[52] Costa AL, Blosi M, Brigliadori A, Zanoni I, Ortelli S, Carlo Simeone F, Delbue S, D’Alessandro S, Parapini S, Vineis C, Varesano A, Toprak S, Hamawandi M, Gardini B. Eco design for Ag-based solutions against SARS-CoV-2 and E. coli. Environ Sci Nano. 2022;9(11):4295–304.

[53] Duman H, Eker F, Akdaşçi E, Witkowska AM, Bechelany M, Karav S. Silver nanoparticles: a comprehensive review of synthesis methods and chemical and physical properties. Nanomaterials. 2024;14(18):1527. 39330683 10.3390/nano14181527 PMC11434896

[54] Fahim M, Shahzaib A, Nishat N, Jahan A, Bhat TA, Inam A. Green synthesis of silver nanoparticles: a comprehensive review of methods, influencing factors, and applications. JCIS Open. 2024;16:100125.

[55] Garibo D, Borbón-Nuñez HA, de León JND, García Mendoza E, Estrada I, Toledano-Magaña Y, Tiznado H, Ovalle-Marroquin M, Soto-Ramos AG, Blanco A, Rodríguez JA, Romo OA, Chávez-Almazán LA, Susarrey-Arce A. Green synthesis of silver nanoparticles using lysiloma acapulcensis exhibit high-antimicrobial activity. Sci Rep. 2020;10(1):12805. 32732959 10.1038/s41598-020-69606-7 PMC7393152

[56] Koduru JR, Kailasa SK, Bhamore JR, Kim KH, Dutta T, Vellingiri K. Phytochemical-assisted synthetic approaches for silver nanoparticles antimicrobial applications: a review. Adv Colloid Interface Sci. 2018;256:326–39. 29549999 10.1016/j.cis.2018.03.001

[57] Ahmed B, Bilal Tahir M, Sagir M, Hassan M. Bio-inspired sustainable synthesis of silver nanoparticles as next generation of nanoproduct in antimicrobial and catalytic applications. Mater Sci Eng B. 2024;301:117165.

[58] Ahmed T, Ogulata RT. A review on silver nanoparticles -green Synthesis, antimicrobial action and application in textiles. J Nat Fibers. 2022;19(14):8463–84.

[59] Ponsanti K, Tangnorawich B, Ngernyuang N, Pechyen C. A flower shape-green synthesis and characterization of silver nanoparticles (AgNPs) with different starch as a reducing agent. J Mater Res Technol. 2020;9(5):11003–12.

[60] Khane Y, Benouis K, Albukhaty S, Sulaiman GM, Abomughaid MM, Al Ali A, Aouf D, Fenniche F, Khane S, Chaibi W, Henni A, Bouras HD, Dizge N. Green synthesis of silver nanoparticles using aqueous citrus Limon zest extract: characterization and evaluation of their antioxidant and antimicrobial properties. Nanomaterials. 2022;12(12):2013. 35745352 10.3390/nano12122013 PMC9227472

[61] Bettini S, Pagano R, Valli L, Giancane G. Drastic nickel ion removal from aqueous solution by curcumin-capped ag nanoparticles. Nanoscale. 2014;6(17):10113–7. 25036541 10.1039/c4nr02583k

[62] Aghajanyan A, Gabrielyan L, Schubert R, Trchounian A. Silver ion bioreduction in nanoparticles using *Artemisia annua* L. extract: characterization and application as antibacterial agents. AMB Express. 2020;10(1):66. 32266590 10.1186/s13568-020-01002-w PMC7138892

[63] González-Ballesteros N, Rodríguez-Argüelles MC, Lastra-Valdor M, González-Mediero G, Rey-Cao S, Grimaldi M, Cavazza A, Bigi F. Synthesis of silver and gold nanoparticles by sargassum muticum biomolecules and evaluation of their antioxidant activity and antibacterial properties. J Nanostruct Chem. 2020;10(4):317–30.

[64] Massironi A, Morelli A, Grassi L, Puppi D, Braccini S, Maisetta G, Esin S, Batoni G, Della Pina C, Chiellini F. Ulvan as novel reducing and stabilizing agent from renewable algal biomass: application to green synthesis of silver nanoparticles. Carbohydr Polym. 2019;203:310–21. 30318218 10.1016/j.carbpol.2018.09.066

[65] Arsyad WS, Cassandra F, Asharuddin M, Suere S, Ramadhan LOAN, Hidayat R. Green synthesis of silver nanoparticles from anthocyanin extracts of purple cabbage (*Brassica oleracea* var capitata) and its characteristics for dye-sensitized solar cells (DSSC) application. J Phys Conf Ser. 2022;2274(1):012001.

[66] Ahmed Mahdi Rheima MAM. Synthesis of silver nanoparticles using the UV-irradiation technique in an antibacterial application. J Southwest Jiaotong Univ [Internet]. 2019 [cited 2022 Dec 14];54(5). http://www.jsju.org/index.php/journal/article/view/392

[67] Su Dlin, Li P jun, Ning M, Li G, yang, Shan Y. Microwave assisted green synthesis of pectin based silver nanoparticles and their antibacterial and antifungal activities. Mater Lett. 2019;244:35–8.

[68] Abeer Mohammed AB, Abd Elhamid MM, Khalil MKM, Ali AS, Abbas RN. The potential activity of biosynthesized silver nanoparticles of *Pseudomonas aeruginosa* as an antibacterial agent against multidrug-resistant isolates from intensive care unit and anticancer agent. Environ Sci Eur. 2022;34(1):109.

[69] Akther T, Khan MohdS SH. Biosynthesis of silver nanoparticles *via* fungal cell filtrate and their anti-quorum sensing against *Pseudomonas aeruginosa*. J Environ Chem Eng. 2020;8(6):104365.

[70] Ferdous Z, Nemmar A. Health impact of silver nanoparticles: a review of the biodistribution and toxicity following various routes of exposure. Int J Mol Sci. 2020;21(7):2375. 32235542 10.3390/ijms21072375 PMC7177798

[71] Rezvani E, Rafferty A, McGuinness C, Kennedy J. Adverse effects of nanosilver on human health and the environment. Acta Biomater. 2019;94:145–59. 31125729 10.1016/j.actbio.2019.05.042

[72] González-Vega JG, García-Ramos JC, Chavez-Santoscoy RA, Castillo-Quiñones JE, Arellano-Garcia ME, Toledano-Magaña Y. Lung models to evaluate silver nanoparticles’ toxicity and their impact on human health. Nanomaterials. 2022;12(13):2316. 35808152 10.3390/nano12132316 PMC9268743

[73] Azizi-Lalabadi M, Garavand F, Jafari SM. Incorporation of silver nanoparticles into active antimicrobial nanocomposites: release behavior, analyzing techniques, applications and safety issues. Adv Colloid Interface Sci. 2021;293:102440. 34022748 10.1016/j.cis.2021.102440

[74] Kose O, Mantecca P, Costa A, Carrière M. Putative adverse outcome pathways for silver nanoparticle toxicity on mammalian male reproductive system: a literature review. Part Fibre Toxicol. 2023;20(1):1. 36604752 10.1186/s12989-022-00511-9 PMC9814206

[75] Sadeghi L, Dantan JY, Siadat A, Marsot J. Design for human safety in manufacturing systems: applications of design theories, methodologies, tools and techniques. J Eng Des. 2016;27(12):844–77.

[76] Zielińska A, Costa B, Ferreira MV, Miguéis D, Louros JMS, Durazzo A, Lucarini M, Eder P, Chaud V, Morsink M, Willemen M, Severino N, Santini P, Souto A. Nanotoxicology and nanosafety: safety-by-design and testing at a glance. Int J Environ Res Public Health. 2020;17(13):4657. 32605255 10.3390/ijerph17134657 PMC7369733

[77] Furxhi I, Faccani L, Zanoni I, Brigliadori A, Vespignani M, Costa AL. Design rules applied to silver nanoparticles synthesis: a practical example of machine learning application. Comput Struct Biotechnol J. 2024;25:20–33. 38444982 10.1016/j.csbj.2024.02.010 PMC10914561

[78] Furxhi I, Bengalli R, Motta G, Mantecca P, Kose O, Carriere M, Haq EU, O’Mahony C, Blosi M, Gardini D, Costa A. Data-driven quantitative intrinsic hazard criteria for nanoproduct development in a safe-by-design paradigm: a case study of silver nanoforms. ACS Appl Nano Mater. 2023;6(5):3948–62. 36938492 10.1021/acsanm.3c00173 PMC10012170

[79] Farjana SH, Huda N, Mahmud MAP. Life cycle analysis of copper-gold-lead-silver-zinc beneficiation process. Sci Total Environ. 2019;659:41–52. 30594860 10.1016/j.scitotenv.2018.12.318

[80] Iravani S, Korbekandi H, Mirmohammadi SV, Zolfaghari B. Synthesis of silver nanoparticles: chemical, physical and biological methods. Res Pharm Sci. 2014;9(6):385–406. 26339255 PMC4326978

[81] Abbasi T, Poornima P, Kannadasan T, Abbasi SA. Acid rain: past, present, and future. Int J Environ Eng [Internet]. 2013 Jun 24 [cited 2023 Mar 10]; https://www.inderscienceonline.com/doi/10.1504/IJEE.2013.054703

[82] Jacobs J, Poel I, Osseweijer P. In. Towards safety and sustainability by design: Nano-sized TiO_2_ in sunscreens. 2010. pp. 187–98.

[83] Abbas R, Luo J, Qi X, Naz A, Khan IA, Liu H, Yu S, Wei J. Silver nanoparticles: synthesis, structure, properties and applications. Nanomaterials. 2024;14(17):1425. 39269087 10.3390/nano14171425 PMC11397261

[84] Ahmad A, Mushtaq Z, Saeed F, Afzaal M, Al Jbawi E. Ultrasonic-assisted green synthesis of silver nanoparticles through cinnamon extract: biochemical, structural, and antimicrobial properties. Int J Food Prop. 2023;26(1):1984–94.

[85] Hoang VT, Dinh NX, Pham TN, Hoang TV, Tuan PA, Huy TQ, Le AT. Scalable electrochemical synthesis of novel biogenic silver nanoparticles and its application to high-sensitive detection of 4-nitrophenol in aqueous system. Adv Polym Technol. 2021;2021(1):6646219.

[86] Nouri A, Tavakkoli Yaraki M, Lajevardi A, Rezaei Z, Ghorbanpour M, Tanzifi M. Ultrasonic-assisted green synthesis of silver nanoparticles using mentha aquatica leaf extract for enhanced antibacterial properties and catalytic activity. Colloid Interface Sci Commun. 2020;35:100252.

[87] Rafique S, Sadiq MB, Akram R, Hussain M, Rizwan M, Bashir M, Khan JS, Awan SU. Facile green microwave-assisted synthesis of silver-chitosan nanoparticle for antioxidant, antibacterial, and non-enzymatic biosensor for dopamine detection. J Mater Res. 2023;38(9):2401–12.

[88] Ashraf H, Anjum T, Riaz S, Naseem S. Microwave-assisted green synthesis and characterization of silver nanoparticles using melia azedarach for the management of fusarium wilt in tomato. Front Microbiol [Internet]. 2020 [cited 2022 Dec 14];11. https://www.frontiersin.org/articles/.10.3389/fmicb.2020.00238 10.3389/fmicb.2020.00238 PMC7076090 32210928

[89] Anh NP, Linh DN, Minh NV, Tri N. Positive effects of the ultrasound on biosynthesis, characteristics and antibacterial activity of silver nanoparticles using *Fortunella japonica*. Mater Trans. 2019;60(9):2053–8.

[90] Abbasi BH, Nazir M, Muhammad W, Hashmi SS, Abbasi R, Rahman L, Hano C. A comparative evaluation of the antiproliferative activity against HepG2 liver carcinoma cells of plant-derived silver nanoparticles from Basil extracts with contrasting anthocyanin contents. Biomolecules. 2019;9(8):320. 31366167 10.3390/biom9080320 PMC6722760

[91] Swilam N, Nematallah KA. Polyphenols profile of pomegranate leaves and their role in green synthesis of silver nanoparticles. Sci Rep. 2020;10(1):14851. 32908245 10.1038/s41598-020-71847-5 PMC7481211

[92] Yu J, Sun L. Facile one-pot synthesis of silver nanoparticles supported on α-zirconium phosphate single-layer nanosheets. ES Mater Manuf. 2019;5(4):24–8.

[93] Zeroual S, Estellé P, Cabaleiro D, Vigolo B, Emo M, Halim W, Ouaskit S. Ethylene glycol based silver nanoparticles synthesized by polyol process: characterization and thermophysical profile. J Mol Liq. 2020;310:113229.

[94] Altemimi A, Lakhssassi N, Baharlouei A, Watson DG, Lightfoot DA. Phytochemicals: extraction, isolation, and identification of bioactive compounds from plant extracts. Plants. 2017;6(4):42. 28937585 10.3390/plants6040042 PMC5750618

[95] Katekar VP, Rao AB, Sardeshpande VR. Review of the Rose essential oil extraction by hydrodistillation: an investigation for the optimum operating condition for maximum yield. Sustain Chem Pharm. 2022;29:100783.

[96] Antony JJ, Sivalingam P, Siva D, Kamalakkannan S, Anbarasu K, Sukirtha R, Krishnan M, Achiraman S. Comparative evaluation of antibacterial activity of silver nanoparticles synthesized using rhizophora apiculata and glucose. Colloids Surf B Biointerfaces. 2011;88(1):134–40. 21764570 10.1016/j.colsurfb.2011.06.022

[97] Kumar B, Smita K, Cumbal L, Debut A. Ficus carica (Fig) fruit mediated green synthesis of silver nanoparticles and its antioxidant activity: a comparison of thermal and ultrasonication approach. BioNanoScience. 2016;6(1):15–21.

[98] Calderón-Jiménez B, Johnson ME, Montoro Bustos AR, Murphy KE, Winchester MR, Vega Baudrit JR. Silver nanoparticles: technological advances, societal impacts, and metrological challenges. Front Chem [Internet]. 2017 Feb 21 [cited 2025 Jan 30]; 5. https://www.frontiersin.org/journals/chemistry/articles/10.3389/fchem.2017.00006/full 10.3389/fchem.2017.00006 PMC5318410 28271059

[99] Hossain MT, Reza S, Islam MA, Md M, Islam T. Progress and prospects of chemical functionalization of textiles via nanotechnology. ACS Appl Eng Mater. 2025;3(1):1–20.

[100] Berton F, Porrelli D, Di Lenarda R, Turco G. A critical review on the production of electrospun nanofibres for guided bone regeneration in oral surgery. Nanomaterials. 2020;10(1):16. 10.3390/nano10010016 PMC7023267 31861582

[101] Saleem H, Zaidi SJ. Sustainable use of nanomaterials in textiles and their environmental impact. Materials. 2020;13(22):5134. 33203051 10.3390/ma13225134 PMC7696606

[102] Agrawal A, Singha K, Pandit P. Methods of application of nanoscale coatings to textiles. In: Ahmed S, Adeel S, editors. Nanoscale textile coatings for enhanced performance [Internet]. Singapore: Springer Nature; 2024 [cited 2025 Feb 19]. pp. 51–79. 10.1007/978-981-97-5922-4_4

[103] Islam Sul, Sun G. Biological chemicals as sustainable materials to synthesize metal and metal oxide nanoparticles for textile surface functionalization. ACS Sustain Chem Eng. 2022;10(31):10084–104.

[104] Perelshtein I, Perkas N, Gedanken A. Ultrasonic coating of textiles by antibacterial and antibiofilm nanoparticles. In: Handbook of ultrasonics and sonochemistry [Internet]. Singapore: Springer; 2016 [cited 2025 Mar 9]. pp. 967–93. 10.1007/978-981-287-278-4_20

[105] Aladpoosh R, Montazer M. Nano-photo active cellulosic fabric through in situ phytosynthesis of star-like Ag/ZnO nanocomposites: investigation and optimization of attributes associated with photocatalytic activity. Carbohydr Polym. 2016;141:116–25. 26877003 10.1016/j.carbpol.2016.01.005

[106] Jiang S, Cui C, Bai W, Wang W, Ren E, Xiao H, Zhou M, Cheng C, Guo R. Shape-controlled silver nanoplates colored fabric with tunable colors, photothermal antibacterial and colorimetric detection of hydrogen sulfide. J Colloid Interface Sci. 2022;626:1051–61. 35868195 10.1016/j.jcis.2022.07.011

[107] Pereira C, Pereira AM, Freire C, Pinto TV, Costa RS, Teixeira JS. Chapter 21 - Nanoengineered textiles: from advanced functional nanomaterials to groundbreaking high-performance clothing. In: Mustansar Hussain C, editor. Handbook of functionalized nanomaterials for industrial applications [Internet]. Elsevier; 2020 [cited 2025 Jan 28]. pp. 611–714. (Micro and Nano Technologies). https://www.sciencedirect.com/science/article/pii/B9780128167878000211

[108] Ojstršek A, Jug L, Plohl O. A review of electro conductive textiles utilizing the dip-coating technique: their functionality, durability and sustainability. Polymers. 2022;14(21):4713. 36365707 10.3390/polym14214713 PMC9654088

[109] Del Secco B, Trabucco S, Ravegnani F, Koivisto AJ, Zanoni I, Blosi M, Ortelli S, Altin M, Bartolini G, Costa AL, Belosi F. Particles emission from an industrial spray coating process using nano-materials. Nanomaterials. 2022;12(3):313. 35159658 10.3390/nano12030313 PMC8838285

[110] Tang X, Yan X. Dip-coating for fibrous materials: mechanism, methods and applications. J Sol-Gel Sci Technol. 2017;81(2):378–404.

[111] Agrawal AM, Pandey P. Scale up of Pan coating process using quality by design principles. J Pharm Sci. 2015;104(11):3589–611. 26202540 10.1002/jps.24582

[112] Girotto C, Rand BP, Steudel S, Genoe J, Heremans P. Nanoparticle-based, spray-coated silver top contacts for efficient polymer solar cells. Org Electron. 2009;10(4):735–40.

[113] Shilo Nesa Sherlin H, Korumilli T, Jagajjanani Rao K. Non-leaching nanoparticle functionalized natural fabrics: a review on durability, environmental impacts, and applications. Nanotechnol Environ Eng. 2025;10(1):7.

[114] Igal K, Zanotti K, Zuin VG, Vazquez P. Sol-gel technology for greener and more sustainable antimicrobial textiles that use silica matrices with C, and ag and ZnO as biocides. Curr Res Green Sustain Chem. 2021;4:100177.

[115] Hong H, Jiang L, Tu H, Hu J, Moon KS, Yan X, Wong C. ping. Rational design and evaluation of UV curable nano-silver ink applied in highly conductive textile-based electrodes and flexible silver-zinc batteries. J Mater Sci Technol. 2022;101:294–307.

[116] Guo X, Yao J, Ji F, Wang R, Hao L. UV curable PUA ink with polymerizable surfactant-enhanced Ag@PPy for fabricating flexible and durable conductive coating on the surface of cotton fabric. Prog Org Coat. 2023;174:107239.

[117] Barhoum A, Pal K, Rahier H, Uludag H, Kim IS, Bechelany M. Nanofibers as new-generation materials: from spinning and nano-spinning fabrication techniques to emerging applications. Appl Mater Today. 2019;17:1–35.

[118] Cay A, Tarakçıoğlu I, Hepbasli A. Assessment of finishing processes by exhaustion principle for textile fabrics: an exergetic approach. Appl Therm Eng. 2009;29(11):2554–61.

[119] Joshi M, Butola BS. 14 - Application technologies for coating, lamination and finishing of technical textiles. In: Gulrajani ML, editor. Advances in the dyeing and finishing of technical textiles [Internet]. Woodhead Publishing; 2013 [cited 2025 Feb 19]. pp. 355–411. (Woodhead Publishing Series in Textiles). Available from: https://www.sciencedirect.com/science/article/pii/B9780857094339500146

[120] Zhang S, Xu C, Xie R, Yu H, Sun M, Li F. Environmental assessment of fabric wet processing from gate-to-gate perspective: comparative study of weaving and materials. Sci Total Environ. 2023;857:159495. 36257424 10.1016/j.scitotenv.2022.159495

[121] Hicks AL, Gilbertson LM, Yamani JS, Theis TL, Zimmerman JB. Life cycle payback estimates of nanosilver enabled textiles under different silver loading, release, and laundering scenarios informed by literature review. Environ Sci Technol. 2015;49(13):7529–42. 26034879 10.1021/acs.est.5b01176

[122] Llewellyn SV, Conway GE, Zanoni I, Jørgensen AK, Shah UK, Seleci DA, Keller JG, Kim JW, Wohlleben W, Jensen KA, Costa A, Jenkins GJS, Clift MJD, Doak SH. Understanding the impact of more realistic low-dose, prolonged engineered nanomaterial exposure on genotoxicity using 3D models of the human liver. J Nanobiotechnol. 2021;19(1):193. 10.1186/s12951-021-00938-w PMC8240362 34183029

[123] Larese FF, D’Agostin F, Crosera M, Adami G, Renzi N, Bovenzi M, Maina G. Human skin penetration of silver nanoparticles through intact and damaged skin. Toxicology. 2009;255(1):33–7. 18973786 10.1016/j.tox.2008.09.025

[124] Larese FF, Mauro M, Adami G, Bovenzi M, Crosera M. Nanoparticles skin absorption: new aspects for a safety profile evaluation. Regul Toxicol Pharmacol. 2015;72(2):310–22. 25979643 10.1016/j.yrtph.2015.05.005

[125] Prasath S, Palaniappan K. Is using nanosilver mattresses/pillows safe? A review of potential health implications of silver nanoparticles on human health. Environ Geochem Health. 2019;41(5):2295–313. 30671691 10.1007/s10653-019-00240-7

[126] Qi M, Wang X, Chen J, Liu Y, Liu Y, Jia J, Li L, Yue T, Gao L, Yan B, Zhao B, Xu M. Transformation, absorption and toxicological mechanisms of silver nanoparticles in the gastrointestinal tract following oral exposure. ACS Nano. 2023;17(10):8851–65. 37145866 10.1021/acsnano.3c00024

[127] Ibrahim SM, El Said A, Hamouda T. Spinning techniques of poly (vinyl Alcohol) fibers for various textile applications. Egypt J Chem. 2024;67(1):447–66.

[128] Bhat MA, Eraslan FN, Gedik K, Gaga EO. Impact of textile product emissions: toxicological considerations in assessing indoor air quality and human health. In: Malik JA, Marathe S, editors. Ecological and health effects of building materials [Internet]. Cham: Springer International Publishing; 2022 [cited 2025 Feb 13]. pp. 505–41. 10.1007/978-3-030-76073-1_27

[129] Roungpaisan N, Srisawat N, Rungruangkitkrai N, Chartvivatpornchai N, Boonyarit J, Kittikorn T, Chollakup R. Melt spinning process optimization of polyethylene terephthalate fiber structure and properties from Tetron cotton knitted fabric. Polymers. 2023;15(22):4364. 38006089 10.3390/polym15224364 PMC10675149

[130] Wu S, Dong T, Li Y, Sun M, Qi Y, Liu J, Kuss MA, Chen S, Duan B. State-of-the-art review of advanced electrospun nanofiber yarn-based textiles for biomedical applications. Appl Mater Today. 2022;27:101473. 35434263 10.1016/j.apmt.2022.101473 PMC8994858

[131] Montes-Hernandez G, Di Girolamo M, Sarret G, Bureau S, Fernandez-Martinez A, Lelong C, Eymard Vernain E. In situ formation of silver nanoparticles (Ag-NPs) onto textile fibers. ACS Omega. 2021;6(2):1316–27. 33490791 10.1021/acsomega.0c04814 PMC7818644

[132] Tang XH, Yan LF, Gao J, Yang XL, Xu YX, Ge HY, Yang HD. Antitumor and Immunomodulatory activity of polysaccharides from the root of limonium Sinense Kuntze. Int J Biol Macromol. 2012;51(5):1134–9. 22960080 10.1016/j.ijbiomac.2012.08.037

[133] PubChem. Sodium borohydride [Internet]. [cited 2025 Feb 13]. https://pubchem.ncbi.nlm.nih.gov/compound/4311764

[134] Nelson AL, Porter L. Hydrogen Peroxide Toxicity. In: StatPearls [Internet]. Treasure Island (FL): StatPearls Publishing; 2023 [cited 2025 Feb 13]. http://www.ncbi.nlm.nih.gov/books/NBK585102/36256749

[135] El-Naggar ME, Khattab TA, Abdelrahman MS, Aldalbahi A, Hatshan MR. Development of antimicrobial, UV blocked and photocatalytic self-cleanable cotton fibers decorated with silver nanoparticles using silver carbamate and plasma activation. Cellulose. 2021;28(2):1105–21.

[136] PubChem. Ditiocarb silver [Internet]. [cited 2025 Feb 14]. https://pubchem.ncbi.nlm.nih.gov/compound/3034078

[137] Weisburger EK. History and background of the threshold limit value committee of the American Conference of Governmental Industrial Hygienists. Chem Health Saf. 2001;8(4):10–2.

[138] NIOSH. Current intelligence bulletin 70: health effects of occupational exposure to silver nanomaterials. 2024 [cited 2025 Jan 30]. https://www.cdc.gov/niosh/docs/2021-112/default.html

[139] Koivisto AJ, Del Secco B, Trabucco S, Nicosia A, Ravegnani F, Altin M, Cabellos J, Furxhi I, Blosi M, Costa A, Lopez de Ipiña J, Belosi F. Quantifying emission factors and setting conditions of use according to ECHA chapter R.14 for a spray process designed for nanocoatings—a case study. Nanomaterials. 2022;12(4):596. 35214925 10.3390/nano12040596 PMC8876979

[140] McCormick S, Niang M, Dahm MM. Occupational exposures to engineered nanomaterials: a review of workplace exposure assessment methods. Curr Environ Health Rep. 2021;8(3):223–34. 34101152 10.1007/s40572-021-00316-6 PMC10079776

[141] Shekaftik SO, Shirazi H, Yarahmadi F, Rasouli R, Ashtarinezhad M. Investigating the relationship between occupational exposure to nanomaterials and symptoms of nanotechnology companies’ employees. Arch Environ Occup Health. 2022;77(3):209–18. 33355040 10.1080/19338244.2020.1863315

[142] Trabucco S, Koivisto AJ, Ravegnani F, Ortelli S, Zanoni I, Blosi M, Costa AL, Belosi F. Measuring TiO_2_N and AgHEC airborne particle density during a spray coating process. Toxics. 2022;10(9):498. 36136463 10.3390/toxics10090498 PMC9503037

[143] West GH, Castaneda FI, Burrelli LG, Dresser D, Cooper MR, Brooks SB, Lippy BE. Occupational exposure risk during spraying of biocidal paint containing silver nanoparticles. J Occup Environ Hyg. 2021;18(6):237–49. 33989130 10.1080/15459624.2021.1910277

[144] Kim J, Yu IJ. National survey of workplaces handling and manufacturing nanomaterials, exposure to and health effects of nanomaterials, and evaluation of nanomaterial safety data sheets. BioMed Res Int. 2016;2016(1):8389129. 27556041 10.1155/2016/8389129 PMC4983336

[145] Lee JH, Kwon M, Ji JH, Kang CS, Ahn KH, Han JH, Yu IJ. Exposure assessment of workplaces manufacturing nanosized TiO_2_ and silver. Inhal Toxicol. 2011;23(4):226–36. 21456955 10.3109/08958378.2011.562567

[146] Lee JH, Ahn K, Kim SM, Jeon KS, Lee JS, Yu IJ. Continuous 3-day exposure assessment of workplace manufacturing silver nanoparticles. J Nanoparticle Res. 2012;14(9):1134.

[147] Li N, Georas S, Alexis N, Fritz P, Xia T, Williams MA, Horner E, Nel A. A work group report on ultrafine particles (American Academy of Allergy, Asthma & Immunology): why ambient ultrafine and engineered nanoparticles should receive special attention for possible adverse health outcomes in human subjects. J Allergy Clin Immunol. 2016;138(2):386–96. 27130856 10.1016/j.jaci.2016.02.023 PMC4976002

[148] Pourzahedi L, Vance M, Eckelman MJ. Life cycle assessment and release studies for 15 nanosilver-enabled consumer products: investigating hotspots and patterns of contribution. Environ Sci Technol. 2017;51(12):7148–58. 28537069 10.1021/acs.est.6b05923

[149] Sarker MSI, Bartok I. Global trends of green manufacturing research in the textile industry using bibliometric analysis. Case Stud Chem Environ Eng. 2024;9:100578.

[150] Roy R, Chavan PP, Rajeev Y, Praveenraj T, Kolazhi P. Sustainable manufacturing practices in textiles and fashion. In: Muthu SS, editor. Sustainable manufacturing practices in the textiles and fashion sector [Internet]. Cham: Springer Nature Switzerland; 2024 [cited 2025 Mar 3]. pp. 1–22. 10.1007/978-3-031-51362-6_1

[151] Leal Filho W, Dinis MAP, Liakh O, Paço A, Dennis K, Shollo F, Sidsaph H. Reducing the carbon footprint of the textile sector: an overview of impacts and solutions. Text Res J. 2024;94(15–16):1798–814.

[152] Pourzahedi L, Eckelman MJ. Comparative life cycle assessment of silver nanoparticle synthesis routes. Environ Sci Nano. 2015;2(4):361–9.

[153] Beigzadeh Z, Kolahdouzi M, Kalantary S, Golbabaei F. A systematic review of released nano-particles from commercial nano-textiles during use and washing. J Ind Text. 2024;54:15280837241254512.

[154] Rujido-Santos I, del Castillo Busto ME, Abad-Alvaro I, Herbello-Hermelo P, Bermejo-Barrera P, Barciela-Alonso MC, Goneaga-Infante H, Moreda-Piñeiro A. spICP-MS characterisation of released silver nanoparticles from (nano)textile products. Spectrochim Acta Part B Spectrosc. 2022;195:106505.

[155] Wagener S, Dommershausen N, Jungnickel H, Laux P, Mitrano D, Nowack B, Schneider G, Luch A. Textile functionalization and its effects on the release of silver nanoparticles into artificial sweat. Environ Sci Technol. 2016;50(11):5927–34. 27128362 10.1021/acs.est.5b06137

[156] Gagnon V, Button M, Boparai HK, Nearing M, O’Carroll DM, Weber KP. Influence of realistic wearing on the morphology and release of silver nanomaterials from textiles. Environ Sci Nano. 2019;6(2):411–24.

[157] Patch D, Koch I, Peloquin D, O’Carroll D, Weber K. Development and validation of a method for the weathering and detachment of representative nanomaterials from conventional silver-containing textiles. Chemosphere. 2021;284:131269. 34186226 10.1016/j.chemosphere.2021.131269

[158] Peloquin DM, Baumann EJ, Luxton TP. Multi-method assessment of PVP-coated silver nanoparticles and artificial sweat mixtures. Chemosphere. 2020;249:126173. 32065993 10.1016/j.chemosphere.2020.126173 PMC7449241

[159] Hedberg J, Eriksson M, Kesraoui A, Norén A, Odnevall Wallinder I. Transformation of silver nanoparticles released from skin cream and mouth spray in artificial sweat and saliva solutions: particle size, dissolution, and surface area. Environ Sci Pollut Res. 2021;28(10):12968–79. 10.1007/s11356-020-11241-w PMC7921047 33097992

[160] Kim JB, Kim JY, Yoon TH. Determination of silver nanoparticle species released from textiles into artificial sweat and laundry wash for a risk assessment. Hum Ecol Risk Assess Int J. 2017;23(4):741–50.

[161] Limpiteeprakan P, Babel S, Lohwacharin J, Takizawa S. Release of silver nanoparticles from fabrics during the course of sequential washing. Environ Sci Pollut Res. 2016;23(22):22810–8. 10.1007/s11356-016-7486-3 27566159

[162] Kraeling MEK, Topping VD, Keltner ZM, Belgrave KR, Bailey KD, Gao X, Yourick JJ. *In vitro* percutaneous penetration of silver nanoparticles in pig and human skin. Regul Toxicol Pharmacol. 2018;95:314–22. 29635060 10.1016/j.yrtph.2018.04.006

[163] Koivisto AJ, Burrueco-Subirà D, Candalija A, Vázquez-Campos S, Nicosia A, Ravegnani F, Furxhi I, Brigliadori A, Zanoni I, Blosi M, Costa A, Belosi F, Lopez de Ipiña J. Exposure assessment and risks associated with wearing silver nanoparticle-coated textiles. Open Res Eur. 2024;4:100. 39639924 10.12688/openreseurope.17254.2 PMC11617820

[164] Olugbodi JO, Lawal B, Bako G, Onikanni AS, Abolenin SM, Mohammud SS, Ataya FS, Batiha GES. Effect of sub-dermal exposure of silver nanoparticles on hepatic, renal and cardiac functions accompanying oxidative damage in male Wistar rats. Sci Rep. 2023;13(1):10539. 37386048 10.1038/s41598-023-37178-x PMC10310751

[165] Moloi MS, Lehutso RF, Erasmus M, Oberholster PJ, Thwala M. Aquatic environment exposure and toxicity of engineered nanomaterials released from nano-enabled products: current status and data needs. Nanomaterials. 2021;11(11):2868. 34835631 10.3390/nano11112868 PMC8618637

[166] Radwan IM, Eljamal O. A mini-review on transportation and fate of silver nanoparticles released from consumer products: ecological risk assessments. Proc Int Exch Innov Conf Eng Sci IEICES. 2022;8:52–61.

[167] Karim N, Afroj S, Lloyd K, Oaten LC, Andreeva DV, Carr C, Farmery AD, Kim ID, Novoselov KS. Sustainable personal protective clothing for healthcare applications: a review. ACS Nano. 2020;14(10):12313–40. 32866368 10.1021/acsnano.0c05537 PMC7518242

[168] Mirzaei M, Furxhi I, Murphy F, Mullins M. Employing supervised algorithms for the prediction of nanomaterial’s antioxidant efficiency. Int J Mol Sci. 2023;24(3):2792. 36769135 10.3390/ijms24032792 PMC9918003

[169] Dutta P, Wang B. Zeolite-supported silver as antimicrobial agents. Coord Chem Rev. 2019;383:1–29.

[170] Fernandes M, Padrão J, Ribeiro AI, Fernandes RDV, Melro L, Nicolau T, Mehravani B, Alves C, Rodrigues R, Zille A. Polysaccharides and metal nanoparticles for functional textiles: a review. Nanomaterials. 2022;12(6):1006. 35335819 10.3390/nano12061006 PMC8950406

[171] Lee D, Sang JS, Yoo PJ, Shin TJ, Oh KW, Park J. Machine-washable smart textiles with photothermal and antibacterial activities from nanocomposite fibers of conjugated polymer nanoparticles and polyacrylonitrile. Polymers. 2019;11(1):16. 10.3390/polym11010016 PMC6402031 30960000

[172] Gao LZ, Bao Y, Cai HH, Zhang AP, Ma Y, Tong XL, Li Z, Dai FY. Multifunctional silk fabric via surface modification of nano-SiO_2_. Text Res J. 2020;90(13–14):1616–27.

[173] Riaz S, Ashraf M, Aziz H, Younus A, Umair M, Salam A, Iqbal K, Hussain MT, Hussain T. Cationization of TiO_2_ nanoparticles to develop highly durable multifunctional cotton fabric. Mater Chem Phys. 2022;278:125573.

[174] Sharkey M, Coggins M. The invisible barrier to safe textile recycling. Front Sustain [Internet]. 2022 [cited 2025 Feb 18]; 3. https://www.frontiersin.org/journals/sustainability/articles/10.3389/frsus.2022.876683/full.

[175] Pensupa N. 12 - Recycling of end-of-life clothes. In: Nayak R, editor. Sustainable technologies for fashion and textiles [Internet]. Woodhead Publishing; 2020 [cited 2025 Feb 18]. pp. 251–309. (Woodhead Publishing Series in Textiles). https://www.sciencedirect.com/science/article/pii/B9780081028674000128

[176] Zhang C, Hu Z, Li P, Gajaraj S. Governing factors affecting the impacts of silver nanoparticles on wastewater treatment. Sci Total Environ. 2016;572:852–73. 27542630 10.1016/j.scitotenv.2016.07.145

[177] Tortella GR, Rubilar O, Durán N, Diez MC, Martínez M, Parada J, Seabra AB. Silver nanoparticles: toxicity in model organisms as an overview of its hazard for human health and the environment. J Hazard Mater. 2020;390:121974. 32062374 10.1016/j.jhazmat.2019.121974

[178] Temizel-Sekeryan S, Hicks AL. Emerging investigator series: calculating size- and coating-dependent effect factors for silver nanoparticles to inform characterization factor development for usage in life cycle assessment. Environ Sci Nano. 2020;7(9):2436–53.

[179] Bonfanti P, Colombo A, Bengalli R, Gualtieri M, Zanoni I, Blosi M, Costa A, Mantecca P. Functional silver-based nanomaterials affecting zebrafish development: the adverse outcomes in relation to the nanoparticle physical and chemical structure. Environ Sci Nano. 2024;11(6):2521–40.

[180] Brunetti G, Donner E, Laera G, Sekine R, Scheckel KG, Khaksar M, Vasilev K, De Mastro G, Lombi E. Fate of zinc and silver engineered nanoparticles in sewerage networks. Water Res. 2015;77:72–84. 25841090 10.1016/j.watres.2015.03.003

[181] Kaegi R, Voegelin A, Sinnet B, Zuleeg S, Hagendorfer H, Burkhardt M, Siegrist H. Behavior of metallic silver nanoparticles in a pilot wastewater treatment plant. Environ Sci Technol. 2011;45(9):3902–8. 21466186 10.1021/es1041892

[182] Wang P, Menzies NW, Dennis PG, Guo J, Forstner C, Sekine R, Lombi E, Kappen P, Bertsch PM, Kopittke PM. Silver nanoparticles entering soils via the Wastewater–Sludge–Soil pathway pose low risk to plants but elevated Cl concentrations increase ag bioavailability. Environ Sci Technol. 2016;50(15):8274–81. 27380126 10.1021/acs.est.6b01180

[183] Doolette CL, McLaughlin MJ, Kirby JK, Navarro DA. Bioavailability of silver and silver sulfide nanoparticles to lettuce (*Lactuca sativa*): effect of agricultural amendments on plant uptake. J Hazard Mater. 2015;300:788–95. 26322966 10.1016/j.jhazmat.2015.08.012

[184] Kaegi R, Voegelin A, Ort C, Sinnet B, Thalmann B, Krismer J, Hagendorfer H, Elumelu M, Mueller E. Fate and transformation of silver nanoparticles in urban wastewater systems. Water Res. 2013;47(12):3866–77. 23571111 10.1016/j.watres.2012.11.060

[185] Blaser SA, Scheringer M, MacLeod M, Hungerbühler K. Estimation of cumulative aquatic exposure and risk due to silver: contribution of nano-functionalized plastics and textiles. Sci Total Environ. 2008;390(2):396–409. 18031795 10.1016/j.scitotenv.2007.10.010

[186] Mohan S, Princz J, Ormeci B, DeRosa MC. Morphological transformation of silver nanoparticles from commercial products: modeling from product Incorporation, weathering through use Scenarios, and leaching into wastewater. Nanomaterials. 2019;9(9):1258. 31491889 10.3390/nano9091258 PMC6781014

[187] Barry D, Barbiero C, Briens C, Berruti F. Pyrolysis as an economical and ecological treatment option for municipal sewage sludge. Biomass Bioenergy. 2019;122:472–80.

[188] Leal Filho W, Ellams D, Han S, Tyler D, Boiten VJ, Paço A, Moora H, Balogun AL. A review of the socio-economic advantages of textile recycling. J Clean Prod. 2019;218:10–20.

[189] Mitrano DM, Limpiteeprakan P, Babel S, Nowack B. Durability of nano-enhanced textiles through the life cycle: releases from landfilling after washing. Environ Sci Nano. 2016;3(2):375–87.

[190] Temizel-Sekeryan S, Hicks AL. Cradle-to-grave environmental impact assessment of silver enabled t-shirts: do nano-specific impacts exceed Non nano-specific emissions? NanoImpact. 2021;22:100319. 10.1016/j.impact.2021.100319 35559976

[191] European Commission. Waste Framework Directive [Internet]. 2018 [cited 2022 Nov 20]. https://environment.ec.europa.eu/topics/waste-and-recycling/waste-framework-directive_en

[192] Bartl A. Chapter 10 - Textiles production and end-of-life management options. In: Letcher TM, editor. Plastic Waste and Recycling [Internet]. Academic Press; 2020 [cited 2025 Jan 28]. pp. 251–79. https://www.sciencedirect.com/science/article/pii/B9780128178805000104

[193] Sánchez Jiménez A, Puelles R, Perez-Fernandez M, Barruetabeña L, Jacobsen NR, Suarez-Merino B, Micheletti C, Manier N, Salieri B, Hischier R, Tsekovska R, Handzhiyski Y, Bouillard J, Oudart Y, Galea KS, Kelly S, Shandilya N, Goede H, Gomez-Cordon J, Jensen KA, van Tongeren M, Apostolova MD, Llopis IR. Safe(r) by design guidelines for the nanotechnology industry. NanoImpact. 2022;25:100385. 35559891 10.1016/j.impact.2022.100385

[194] Sánchez Jiménez A, Puelles R, Pérez-Fernández M, Gómez-Fernández P, Barruetabeña L, Jacobsen NR, Suarez-Merino B, Micheletti C, Manier N, Trouiller B, Navas JM, Kalman J, Salieri B, Hischier R, Handzhiyski Y, Apostolova MD, Hadrup N, Bouillard J, Oudart Y, Merino C, Garcia E, Liguori B, Sabella S, Rose J, Masion A, Galea KS, Kelly S, Štěpánková S, Mouneyrac C, Barrick A, Châtel A, Dusinska M, Rundén-Pran E, Mariussen E, Bressot C, Aguerre-Chariol O, Shandilya N, Goede H, Gomez-Cordon J, Simar S, Nesslany F, Jensen KA, van Tongeren M. Rodríguez Llopis I. Safe(r) by design implementation in the nanotechnology industry. NanoImpact. 2020;20:100267.

[195] Trump BD, Keisler JM, Galaitsi SE, Palma-Oliveira JM, Linkov I. Safety-by-design as a governance problem. Nano Today. 2020;35:100989.

[196] Li S, Barnard AS. Safety-by-design using forward and inverse multi-target machine learning. Chemosphere. 2022;303:135033. 35618055 10.1016/j.chemosphere.2022.135033

[197] Furxhi I, Perucca M, Koivisto AJ, Bengalli R, Mantecca P, Nicosia A, Burrueco-Subirà D, Vázquez-Campos S, Lahive E, Blosi M, de Ipiña JL, Oliveira J, Carriere M, Vineis C, Costa A. A roadmap towards safe and sustainable by design nanotechnology: implementation for nano-silver-based antimicrobial textile coatings production by ASINA project. Comput Struct Biotechnol J. 2024;25:127–42. 39040658 10.1016/j.csbj.2024.06.013 PMC11262112

